# Lysophosphatidic acid-induced Arf6-driven macropinocytosis of CD147^+^ extracellular vesicles promotes sorafenib resistance of hepatocellular carcinoma

**DOI:** 10.7150/ijbs.125483

**Published:** 2026-01-01

**Authors:** Luomeng Qian, Zhiguang Fu, Ping Chen, Yuan Guo, Yutong Li, Yang Wang, Bo Wang, Qing Zhang, Qingjun Guo, Lidi Wu, Paulina Kucharzewska, Zhesheng Chen, Yongjun Piao, Sihe Zhang

**Affiliations:** 1 Department of Cell Biology, School of Medicine, Nankai University, Tianjin, 300071, China.; 2 Department of Tumor Radiotherapy, Air Force Medical Center, People's Liberation Army of China (PLA), Beijing, China.; 3 National Clinical Research Center for Cancer, Key Laboratory of Cancer Prevention and Therapy, Tianjin Medical University Cancer Institute and Hospital, Tianjin, 300060, China.; 4 Department of Transplant Surgery, Tianjin First Central Hospital, Tianjin 300192, China.; 5 Center of Cellular Immunotherapies, Warsaw University of Life Sciences, 02-787 Warsaw, Poland.; 6 Department of Pharmaceutical Sciences, College of Pharmacy and Health Sciences, St. John's University, Queens, New York, NY, 11439, USA.

**Keywords:** Extracellular vesicles, Macropinocytosis, Lysophosphatidic acid, Sorafenib resistance, Hepatocellular carcinoma

## Abstract

**Background**: Transarterial chemoembolization (TACE) combined with sorafenib is a common therapeutic strategy for hepatocellular carcinoma (HCC). However, sorafenib resistance (SFR) remains a major clinical obstacle. Evidence suggest that TACE reshapes the tumor microenvironment (TME), creating an external high-glucose (HG) and internal low-glucose (LG) niche. In this context, hyperglycemia-driven lysophosphatidic acid (LPA) production accelerates HCC progression. Moreover, intercellular communication via extracellular vesicles (EVs) has been linked to drug resistance. Despite these insights, the SFR mechanism by which HG-induced LPA regulates EV uptake and signaling is unclear.

**Methods**: ELISA, immunohistochemistry, Western blot, CCK-8, Annexin V-7AAD, bioinformatics, and hyperglycemic models were performed to assess the HG-LPA-EV connection in cell, blood, and surgical samples. Nanoparticle characterization, confocal imaging, GST pull-down, dominant mutants, and UEA-1 blot were used to check Arf6 activation, CD147 fucosylation, and EV-stimulated signaling. Bilateral CDX models, GFP-CD63 imaging, and combinational treatments were performed to further elucidate the SFR mechanism.

**Results**: SFR emerges in hyperglycemic HCC patients with elevated LPA levels. Mechanistically, HG-induced LPA elevation promotes the uptake of LG-derived EVs (LG-EVs), thereby driving resistance. LPA activates ADP-ribosylation factor 6 (Arf6), which enhances macropinocytosis-mediated LG-EV uptake. Further, LG conditions increase fucosyltransferase 1 (FUT1)-dependent CD147 fucosylation on EV surfaces. Uptake of CD147⁺ LG-EVs subsequently promotes SFR by activating the fucosylation-dependent AKT/mTOR/4EBP1 signaling pathway. Importantly, inhibition of LPA-Arf6-mediated EV macropinocytosis significantly improves the sorafenib efficacy.

**Conclusion**: Our findings uncover a previously unrecognized mechanism mediated by differential TME and CD147⁺ EV macropinocytosis in HCC and highlight the LPA-Arf6-macropinocytosis as a novel targeting axis to overcome SFR in HCC.

## Introduction

Hepatocellular carcinoma (HCC), a leading cause of cancer-related mortality, is strongly associated with viral hepatitis, alcohol consumption, and metabolic disorders. Surgical resection and liver transplantation remain the preferred treatments for early-stage HCC. In contrast, advanced HCC patients are typically treated with transarterial chemoembolization (TACE) in combination with targeted therapies 1. Sorafenib has long been the first-line targeted drug for HCC, supported by substantial clinical evidence. However, most HCC patients acquire sorafenib resistance (SFR) within six months following TACE 2. The mechanisms driving SFR are diverse yet remain incompletely understood, underscoring the need for clarification.

Hostile tumor microenvironment (TME) is a critical contributor to the therapeutic resistance of HCC 3. In addition to hypoxia, TACE-induced ischemic stress further amplifies SFR 4. Low glucose (LG) content exists in intratumoral interstitial fluid (0.12 g/L) compared with aortic serum (1.72 g/L) 5. Embolization exacerbates this shortage, and glucose deprivation enhances cancer stemness and drives multidrug resistance (MDR) 6. Paradoxically, epidemiological data indicate that hyperglycemia frequently occurs in post-TACE patients 7. Diabetic patients are prone to postoperative hyperglycemia 8, 9. Even non-diabetic HCC patients with viral infection display higher circulating glucose levels (HG), which correlate with increased risk of disease progression 10. Notably, hyperglycemia is also linked to activated autotaxin (ATX)-lysophosphatidic acid (LPA) signaling axis, which promotes MDR 11, 12. Increased LPA signaling has been implicated in resistance to sunitinib and proteasome inhibitors 13, 14. Thus, a causal relationship between HG-driven ATX-LPA activation and SFR in HCC is strongly suggested.

Increasing evidence support that intercellular transmission of extracellular vehicles (EVs) promotes SFR by modulating anti-apoptotic signaling, immunosuppression, and cancer stemness 15. Although EV cargo contributes to drug resistance, the mechanisms governing EV uptake in HCC and the identity of functional surface molecules remain poorly understood. Known pathways for EV uptake include clathrin-mediated endocytosis (CME), caveolin-dependent endocytosis (CDE), and macropinocytosis 16. Among these, macropinocytosis predominates in cancer cells and can be activated by ADP-ribosylation factor 6 (Arf6) 17, 18. Notably, LPA has been shown to stimulate macropinocytosis in *Entamoeba histolytica*, while glucose supplementation enhances macropinocytosis in *Dictyostelium* 19, 20. More importantly, in HCC, EVs are enriched with microRNAs and frequently defined by glycoprotein CD147 21. Arf6-driven CD147 trafficking contributes to HCC malignancy 22, and CD147 overexpression is evidenced to promote MDR 23. Moreover, glucose deprivation induces aberrant CD147 glycosylation, which enhances cancer stemness 6, 24. These findings suggest a model in which TACE accentuates a differential TME (extratumoral HG vs. intratumoral LG). HG-induced LPA elevation activates Arf6-driven macropinocytosis in peripheral HCC tissue, facilitating CD147^+^ LG-derived EVs (LG-EVs) uptake, thereby promoting SFR.

In this study, we demonstrate that SFR is associated with hyperglycemia and elevated LPA in HCC patients. Mechanistically, HG-induced LPA elevation enhances LG-EV uptake, while LPA-activated Arf6 stimulates macropinocytosis. LG stress increases fucosyltransferase 1 (FUT1)-mediated fucosylation of CD147 on EVs, enabling CD147⁺ LG-EVs to promote SFR through activation of AKT/mTOR/4EBP1 signaling. Most importantly, blocking LPA-induced Arf6-driven EV macropinocytosis significantly improves the sorafenib efficacy. Collectively, our findings uncover a novel SFR mechanism driven by the differential TME and mediated through EV macropinocytosis. The LPA-Arf6-macropinocytosis axis emerges as a promising therapeutic target to overcome SFR.

## Material and methods

### Cell culture, plasmids, antibodies, and chemical reagents

Human HCC cells, HepG2 and MHCC-97H, were obtained from the Type Culture Collection of the Chinese Academy of Sciences (China). Cells were routinely cultured in complete DMEM medium with high glucose (HG, 4.5g/L) or low glucose (LG, 0.45g/L).

Lentiviral plasmid pLV-H1-EF1α-GFP-Puro (SORT-B19) and pLV-EF1α-MCS-IRES-Bsd (pLV03) were purchased from BiOSETTIA. The human wild-type (wt), constitutively active (Q67L), and constitutively inactive (T27N) Arf6-HA/pcDNA3 plasmids were originally generated in our laboratory 18, 22. GGA3-GAT (141-327aa)/pGEX4T-2 plasmid was supplied by Prof. Hiroyuki Takatsu 25. Lentiviral plasmid pLV-EF1α-MCS-EGFP-CD63-IRES-puro was supplied by Prof. Shijing Yue (Nankai University).

Mouse anti-HSP70 Ab (sc-59569) was purchased from Santa Cruz. Rabbit anti-CD63 Ab (R23851), anti-TSG101 Ab (R25999), anti-Calnexin Ab (340144), anti-AKT Ab (R23411), anti-mTOR Ab (380411), anti-4EBP1 Ab (R24197), anti-phospho-mTOR Ab (R381548), anti-phospho-4EBP1 Ab (R22929), anti-Autotaxin (ATX) Ab (252255), goat anti-mouse Ab-HRP (511103), anti-rabbit Ab-HRP (511203), anti-mouse Ab-FITC (511101), and anti-rabbit Ab-FITC (511201) were purchased from ZENBIO. Rabbit anti-Arf6 Ab (20225-1-AP), anti-FUT1 Ab (17956-1-AP), anti-phospho-AKT Ab (66444-1-lg), and mouse anti-GAPDH Ab (60004-1-Ig) were purchased from Proteintech. Mouse anti-GFP Ab (M20004) was purchased from Abmart. Mouse anti-CD147 Ab (H18) was originally produced in our lab 22, 26-28. Biotin-labeled anti-CD147 Ab (E-AB-F1056B) was purchased from Elabscience.

Streptavidin beads (PuriMag^TM^G) were purchased from PuriMag Biotech. Biotin-labeled UEA-1 Lectin (GTX01511) was purchased from Vector Laboratories. Streptavidin-HRP (K1229) was purchased from APEBIO. Sorafenib (S45467) was purchased from Yuanye Biotechnology. LPA (O863560), Wortmannin (681675), and Genistein (G810424) were purchased from MACKLIN. EIPA (E10484) was purchased from SAITONG. Chlorpromazine (CPZ, HY-12708) was purchased from MCE. Rhodamine-Dextran (R8881) and CTxB-FITC (C1655) were purchased from SIGMA. Transferrin-Alexa Fluor 647 (AF647-Tfn, T23366) was purchased from Life Science. Streptozotocin (STZ, 60750ES76) was purchased from YEASEN. Lipofectamine 2000 (Lipo2K, GK20005) was purchased from GLPBIO. DiO (D5840), DiI (D8700), and CCK8 Kit (CA1210) were purchased from Solarbio. Annexin V/7-AAD apoptosis Kit (A5001-02A-L) was purchased from SIMUBIOTECH. LPA ELISA Kit (ml062890) was purchased from Mlbio. An immunohistochemical kit (PV-9000) was purchased from ZSGB-BIO.

### Knockdown and overexpression of target genes

Two independent Arf6-targeting shRNAs (Supplementary Table S1) were generated. The gene silencing system (SORT-B19) was utilized to produce stable HCC cell lines with Arf6-knockdown (Arf6-KD). Arf6 (wt)-, Arf6 (Q67L)-, and Arf6 (T27N)-HA/pcDNA3 plasmids were respectively used as templates to amplify cDNA for Arf6-overexpression (Arf6-OE). The primers listed in Supplementary Table S1 were used. The gene expression system (pLV03) was used to produce HCC cell lines with Arf6-OE. The production and infection of recombinant lentivirus were performed as previously described 18, 22, 27.

The synthesis, Lipo2K-based transfection of CD147- and FUT1-targeted siRNA duplexes (Supplementary Table S1) into the HepG2 cells and MHCC-97H cells was performed as previously described 29-32. Specific knockdown of target proteins was confirmed by western blot using primary Abs followed by corresponding secondary HRP-Abs.

### Isolation of EVs

A differential centrifugation approach was used to isolate EVs from LG-cultured, 75% confluent HCC cells (LG-EVs). Briefly, floating cells and debris were first removed from culture supernatant by centrifuging at 300 × g for 10 minutes. The cell-free supernatant was centrifuged at 2000 × g for 30 minutes, and the harvested supernatant was then ultracentrifuged at 130,000 × g for 1 h. After discarding the supernatants, the pellet was resuspended in PBS, followed by centrifuging at 130,000 × g for 1 hour. The final pellet (EVs) was resuspended in PBS, and the protein concentration of the EV lysate was determined by the standard BCA protein kit.

To isolate CD147⁺ LG-EVs, LG-EVs were first extracted as described above. Streptavidin-coated magnetic beads (10 μg) were incubated with biotin-labeled anti-CD147 antibody (10 μg) overnight at 4 °C. After separation on a magnetic stand, the antibody-conjugated beads were collected and incubated with LG-EVs for 16 hours at 4 °C. Following magnetic separation, the supernatant contained CD147⁻ LG-EVs, while CD147⁺ LG-EVs were recovered from the antibody-conjugated beads by washing. The same procedure was applied to isolate CD147⁺FUT1⁺ LG-EVs and CD147⁺FUT1⁻ LG-EVs, using wild-type and FUT1-knockdown HCC cells, respectively.

### Identification of EVs

Conventional FACS-based analysis of EV surface markers was performed as previously reported 33. Briefly, EVs (1.6 μg/μL) were first labeled with biotin (0.2 μg/μL) at 4 ℃ for 30 minutes in the dark. The streptavidin magnetic beads (10 μg) were diluted in binding buffer and mixed with EVs at 4 ℃ for 1 hour of binding. After absorbing on the magnetic stand, the EV-bound streptavidin-biotin beads were harvested by washing with binding buffer. Then, primary Abs (anti-CD147, 1:2000; anti-CD63, 1:1000; anti-HSP70, 1:1000; anti-TSG101, 1:1000; anti-Calnexin, 1:1000) and FITC-conjugated secondary Abs were sequentially added, followed by incubating with collected EVs-bound streptavidin-biotin-beads at 4 ℃ for 1 hour. After absorbing on a magnetic stand and washing again, the bead-loaded streptavidin-biotin-conjugated EVs were collected and analyzed with flow cytometry.

The morphology of isolated EVs was checked by transmission electron microscopy (TEM) as previously described 29, 32, 34. Briefly, suspended EV suspensions were mounted on copper grids for 5 minutes. Excess fluid was discarded, and EV particles were stained with phosphotungstic acid for two minutes. The remaining staining solution was removed, and the grids were washed with ddH2O, followed by air-drying. EV samples were imaged by JEM-1400 TEM at a voltage of 120 kV.

The size distribution of isolated EVs was analyzed by the particle tracking analyzer (Metrix Zetaview nanoparticle). Data was collected over 2 minutes for each sample.

The expression of EV-specific markers was further determined by Western blotting analysis.

### Cellular viability assay

HCC cells were seeded at a density of 1x10^4^/ml in 96-well plates and grown for 24 h. Sorafenib at IC50 (4 μg/mL for HepG2 and 6 μg/mL for MHCC-97H) was added for 36 hours, with or without EV coculture (2 μg/well) for 24 hours. Enhanced CCK8 solution was further incubated with cells for 1 h at 37 °C. Absorbance was monitored by a microplate reader (450 nm), and cell viability was calculated.

### Cellular apoptosis assay

Apoptosis of HCC cells was measured by the Annexin V/7-AAD apoptosis detection kit according to the manufacturer's instructions. Briefly, HCC cells were pretreated with sorafenib at IC50 (4 μg/mL for HepG2 and 6 μg/mL for MHCC-97H) for 36 hours, or cocultured with EVs (2 μg/well) for 24 hours. Cells were carefully harvested, washed twice with PBS, and stained with Annexin V and 7-AAD diluent for 20 minutes and subjected to analysis using flow cytometry. Data were recorded from 1 × 10^4^ cells in triplicate per condition.

### ELISA assay

Peripheral blood of HCC patients, lysate of HCC tissues, and culture supernatant of HCC cells were centrifuged and collected. The LPA level in these samples was measured by an ELISA Kit according to the manufacturer's instructions.

### EV-labeling and colocalization analysis

EV samples were mixed with fluorescent dyes (DiO or DiI) solution in the dark for 30 min. Unlabeled dyes were removed by centrifuging the mixture at 20000 g for 30 min, and the labeled EV pellet (DiO-EVs, DiI-EVs) was resuspended in PBS. To determine the EV uptake pathway, RhoB-dextran (70 kDa, macropinocytosis maker, 4 mg/mL), AF647-Tfn (CME marker, 25 μg/mL), and FITC-CTxB (CDE marker, 10 μg/mL) were respectively co-incubated with DiO- or DiI-labelled EVs (50 μg/mL) in HCC cells at 37 °C for 1h. After quick-rinsing with PBS/0.5M NaCl, fixing with 4% paraformaldehyde, nuclei-staining with DAPI, HCC cells were immediately image-acquired using a confocal microscope (Olympus FV 1000). The images were processed by ImageJ software, and the co-localization coefficient was calculated.

### EV uptake assay

Cellular uptake of EVs was measured by confocal imaging as previously described 17, 35. HCC cells growing in chamber slides were first starved with serum-free medium for 16 h, then incubated with DiO-EVs or DiI-EVs (50 μg/mL) at 37 °C for 1 hour. To inhibit the uptake of TMR-dextran or DiO-EVs, HCC cells were pretreated with different concentrations of EIPA (0 μM, 25 μM, and 50 μM), wortmannin (0 μM, 0.25 μM, and 0.5 μM), genistein (0 μM, 100 μM, and 200 μM), and CPZ (0 μM, 5 μM, and 10 μM) for 4 hours. These drugs were made in DMSO, diluted in PBS, and the final concentration of DMSO was less than 0.4%. After quick-rinsing, nuclei-staining, and EV uptake imaging by confocal as described above, the total particle per cell area was evaluated from at least five fields.

### Arf6 activation assay

GST-GGA pull-down was used to determine the activation level of Arf6 GTPase as previously described 25. Briefly, serum-starved cells were stimulated with LPA for 30 min, lysed with pull-down solution at 4 °C for 10 min, and incubated with glutathione-sepharose beads immobilized with GST-GGA3 at 4 °C for 1 hour. After subjecting the collected pellets to SDS-PAGE, the GTP-bound Arf6 in the samples was determined by Western blotting.

### Western blotting and UEA1-blotting

HCC cells were lysed in RIPA buffer. The samples were quantified by a BCA kit, resolved by SDS-PAGE, transferred onto PVDF membranes, blotted respectively with primary Abs as following (mouse anti-CD147 Ab, 1:2000; anti-HSP70 Ab, 1:800; anti-AKT Ab, 1:2000; anti-GFP Ab, 1:1000; Rabbit anti-CD63 Ab, 1:1000; anti-TSG101 Ab, 1:1000; anti-FUT1 Ab, 1:1000; anti-Calnexin Ab, 1:1000; anti-mTOR Ab, 1:1000; anti-4EBP1 Ab, 1:1000; anti-p-AKT Ab, 1:1000; anti-p-mTOR, 1:1000; anti-p-4EBP1 Ab, 1:1000), and finally detected with corresponding HRP-labeled secondary Abs.

For the immunoprecipitation (IP)-based UEA1 blot, the cell lysate was first pre-cleared with protein A/G for 1 h. The supernatant was incubated with anti-CD147 Ab (2 μg/200 μg total protein) overnight at 4°C, and then incubated with protein A/G for additional 4 hours. After washing the beads, the collected immunoprecipitation was forwarded to Western blot analysis by biotin-labeled UEA-1 Lectin (1:500), followed by Streptavidin-HRP Ab (1:2000) incubation.

### HCC-xenografted mouse models

A hyperglycemic model was induced by injecting mice with a dose of streptozotocin (STZ) that does not cause diabetes in chow-fed mice 36. Briefly, NOD/SCID mice (female, 6-8 weeks old, Beijing SiPeiFu) were randomly grouped and injected intraperitoneally (i.p.) with STZ solution (50 mg/kg, once a day) for 8 days. Mice in the normoglycemic group (control) were injected (i.p.) with an equal volume of citrate buffer (pH 4.5). Blood glucose concentration was monitored based on the tail-vein blood. STZ-injected mice with 8.3 mmol/L were considered hyperglycemic.

HCC cell-derived xenograft (CDX) models were further created as previously described 26, 37. Briefly, HCC cells (1 × 10^7^) were subcutaneously injected into the left and right armpits of hyperglycemic or normoglycemic NOD/SCID mice. Tumor growth was monitored once every two days by measuring the diameter of tumors with a caliper, and tumor volumes were calculated according to the formula: Volume = (D × d^2^/2), where D is the longest axis of the tumor and d is the shortest length of a prolate ellipse. When the tumor volume reached approximately 0.4 cm in diameter, all mice were randomly grouped (n=5/group) and administered with sorafenib (40 mg kg^-1^) intragastrically every two days. NAV2729 (20 mg kg^-1^), EIPA (10 mg kg^-1^), and LPA (6 mg kg^-1^) were intraperitoneally administered twice a week. Partial mice were imaged under anesthesia by an IVIS Lumina II *in vivo* imaging system (Xenogen) at the indicated time points. The fluorescence intensity of images is reported as photons per second per centimeter squared per steradian (p/s/cm^2^/sr).

### HCC patient samples and immunohistochemistry

Peripheral bloods of HCC patients (normoglycemia vs hyperglycemia) and healthy individuals were collected from the Tianjin Medical University Cancer Institute & Hospital and Tianjin First Central Hospital (China). Fresh tissues and paracancerous tissues (PNT) were collected from HCC patients with hepatectomy and had not undergone radiotherapy before the operation. Each tissue block was sliced (diameter: 0.6 mm), followed either by fixation with 4% paraformaldehyde (for IHC staining) or by freezing with 0.1 M phosphate buffer (for ELISA). Slices of embedded tissues were mounted on glass slices after being sliced into 6 μm sections.

Immunohistological analysis was performed as described previously 26, 27, 37-39. Briefly, after deparaffinization, rehydration, and antigen-retrieval treatment with Tris-EDTA buffer (pH 9.0) at 121 °C for 5 min, slides were treated with hydrogen peroxide to quench endogenous peroxidase, blocked with goat serum, and incubated with primary antibodies (rabbit anti-ATX, 1:200; anti-Arf6 Ab, 1:100. anti-FUT1 Ab, 1:200; mouse anti-CD147 Ab, 1:200; anti-GFP, 1:100.) overnight at 4 °C. Non-immune rabbit or mouse IgG was used as a negative control. IHC staining was performed with the EnvisionTM two-step system (Dako, USA). Slides were treated with 3, 3-diaminobenzidine and counterstained with hematoxylin. Immunopositivity was independently assessed and scored by two pathologists, who were blinded to the clinical data.

### Bioinformatics analysis

Bioinformatics analyses were performed similarly as described previously 22, 27, 29, 30, 38-41. The GEO (https://www.ncbi.nlm.nih.gov/gds) database was employed to analyze the mRNA transcriptional level of ATX, Arf6, FUT1, and CD147 in HCC tissues (GSE109211) and cell lines (GSE128686). Expression-upregulated genes in SFR HCC cells relative to SFS cells were identified by DESeq2 with absolute fold change >2 and adjusted Padj value <0.05. All the fold change data were transformed as log2 (value), and the Padj data were transformed as -log10 (value). The data of log2 (value) > 0 and -log10 (value) > 0 were identified as upregulated genes. The TCGA (https://portal.gdc.cancer.gov/) database was employed to analyze the relationship between sorafenib-IC50 and ATX expression. The HCC patient cohort was divided into IC50-low and IC50-high groups, followed by overall survival (OS) analysis, and the log-rank test was used to examine the differences in OS. Correlation between IC50 value and ATX was further analyzed. The Human Protein Atlas (http://www.proteinatlas.org/) database was employed to analyze the tissue expression level of ATX.

### Statistical analysis

All the results are presented as mean ± SEM values. Statistical analyses were executed with GraphPad Prism (V8.3.0) by using one- or two-way ANOVA or Student's *t* test. Significant difference was defined as * *P* < 0.05, ** *P* < 0.01, and ns: no significant difference. Three independent assays were performed for each methodology unless otherwise stated.

### Study approval

Animal experiments were approved by the Nankai University Animal Care and Use Committee (2021-SYDWLL-000479). Human blood samples were obtained from health examiners, and surgical specimens were obtained from HCC patients (informed consent was obtained from the Review Board of Nankai University, NKUIRB2021043).

## Results

### SFR occurs in hyperglycemic HCC patients with high LPA levels

**Figure 1:**
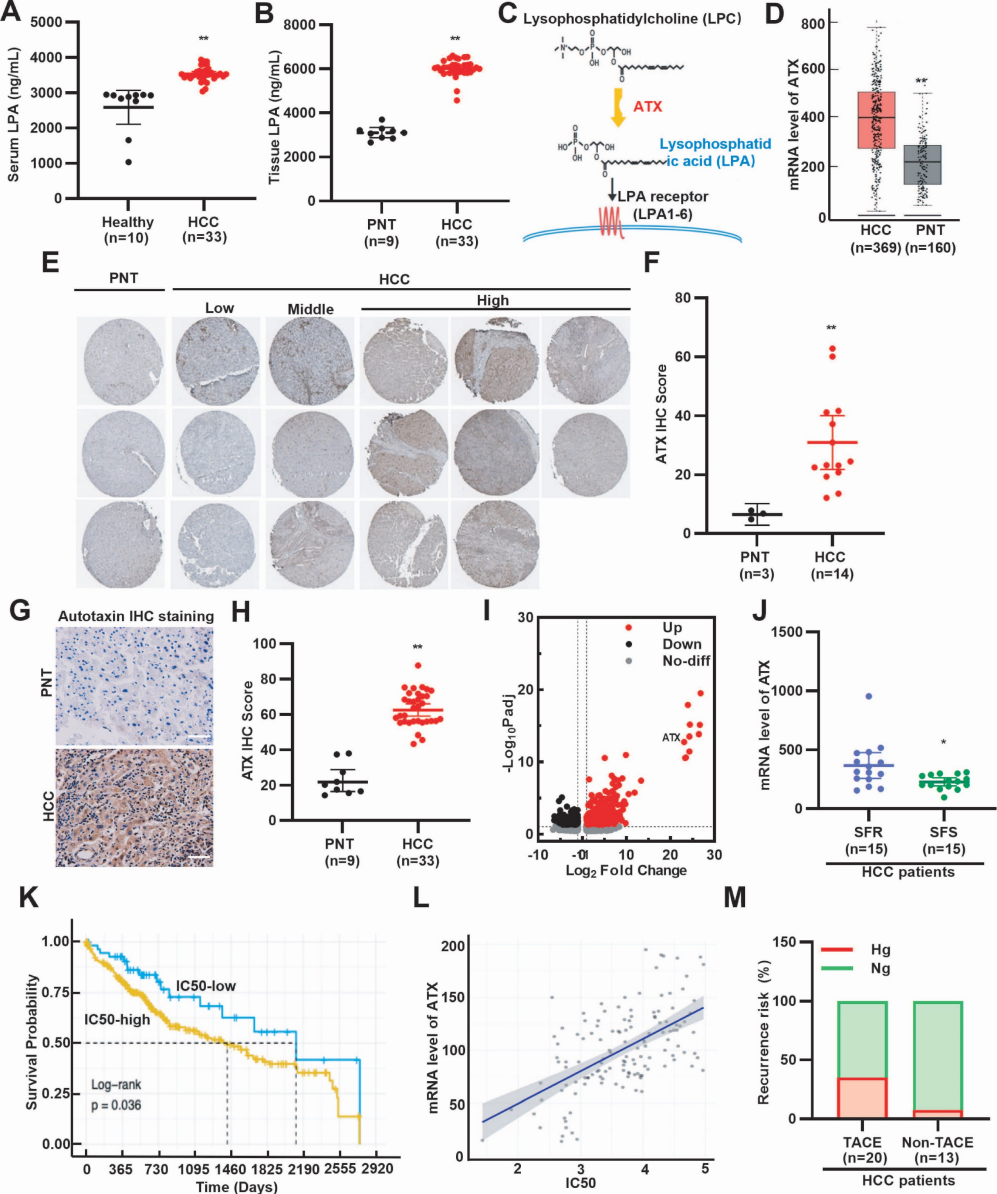
High level of LPA and hyperglycemia is associated with SFR of HCC. (**A**) Serum LPA levels of healthy individuals and HCC patients, (**B**) LPA levels of PNT and HCC tissues were determined by ELISA. (**C**) A diagram illustrating ATX as the key anabolic enzyme producing LPA. (**D**) Box plots show the mRNA expression levels of ATX between HCC and PNT tissues, based on data from the GEO database (GSE102079). (**E**) IHC staining and (**F**) quantitative data of ATX. Data are derived from the HPA database. (**G**) IHC staining and (**H**) quantitative data of ATX in PNT and HCC tissues. Scale bars, 100 µm. (**I**) *ATX* expression is upregulated in SFR-HCC cells. Data are derived from the GEO database (GSE128686). (**J**) mRNA level of *ATX* in SFR- and SFS-HCC tissues. Data are derived from the GEO database (GSE109211). (**K**) OS is shorter in HCC patients treated with IC50-high sorafenib. (**L**) Scatterplot showing positive correlation between SFR (represented by IC50 values) and *ATX* expression. (**M**) Rates of hyperglycemia (Hg, >7.8 mmol/L) and normoglycemia (Ng, ≤7.8 mmol/L) in HCC patients with or without TACE. Recurrence risk (%) means the recurrence rate of hyperglycemia or normoglycemia HCC patients treated with or without TACE. All data are presented as means ± SEM. * *P* < 0.05, ** *P* < 0.01. Student's *t*-test.

To assess whether LPA levels are elevated in HCC patients, we analyzed peripheral blood and surgical specimens. ELISA analysis revealed higher serum LPA concentrations in HCC patients compared with healthy individuals (Fig. 1A). Consistently, LPA levels were higher in HCC tissues than in paired paracancerous tissues (PNT) (Fig. 1B). Further ELISA analysis revealed that sorafenib treatment reduced both serum and tissue LPA levels in HCC patients (Supplementary Fig. S1A, S1B). Since ATX catalyzes the conversion of lysophosphatidylcholine to LPA (Fig. 1C), we next examined ATX expression. GEO and HPA database mining showed that ATX mRNA and protein are upregulated in HCC tissues (Fig. 1D-F). Immunohistochemistry further confirmed elevated ATX expression in patient-derived HCC tissues (Fig. 1G, 1H). These results suggest that LPA levels are elevated in serum and cancer tissues of HCC patients. To determine whether elevated LPA levels are associated with SFR, we examined ATX expression in resistant models. GEO database mining revealed higher ATX expression in SFR-HCC cells and tissues (Fig. 1I, 1J). Survival analysis further showed that sorafenib-treated patients with high ATX expression had shorter OS (Fig. 1K, 1L). These findings suggest that LPA elevation is associated with poor response to sorafenib. Given that hyperglycemia is a known adverse factor for HCC prognosis 7, we next investigated the recurrence risk of HCC patients with or without TACE (Supplementary Table S2). As anticipated, hyperglycemic patients exhibited higher post-TACE relapse (Fig. 1M). Collectively, clinical and bioinformatics data demonstrate that SFR of HCC is strongly associated with hyperglycemia and elevated LPA levels.

### HG-induced LPA elevation promotes LG-EV uptake and SFR

**Figure 2:**
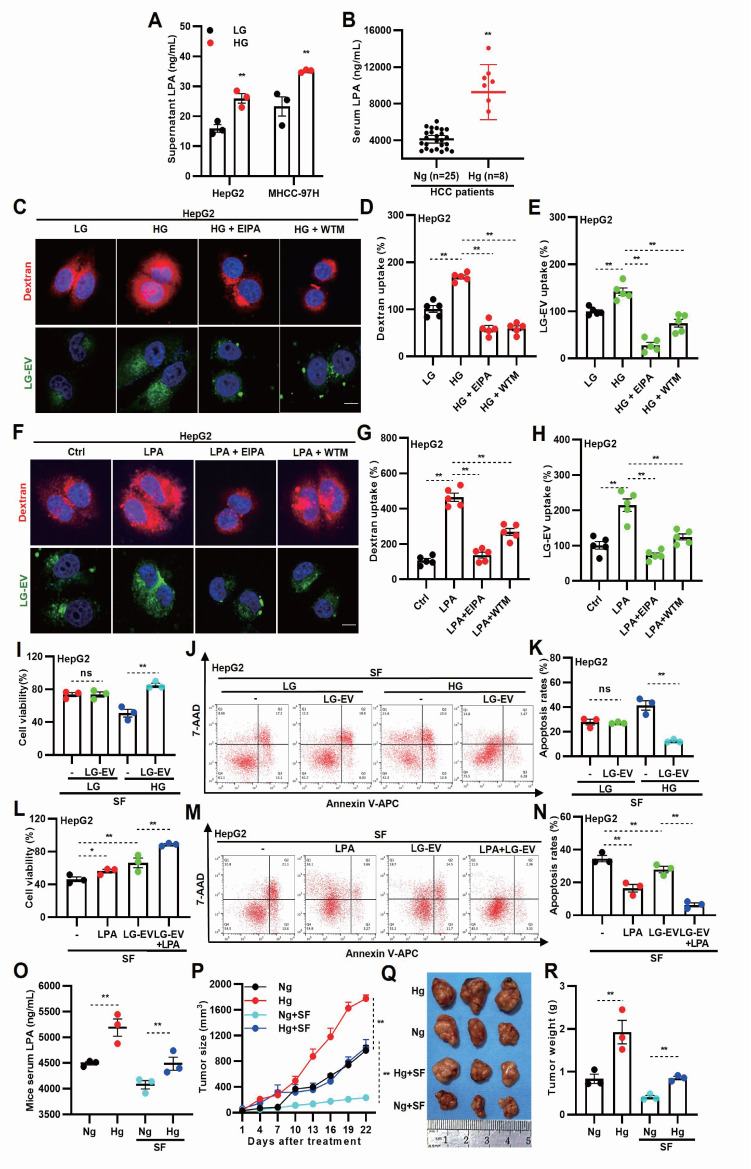
HG-induced LPA elevation promotes LG-culture-derived EV (LG-EV) uptake and SFR. (**A**) LPA level in LG- (0.45 g/L) and HG-(4.5 g/L) culture supernatant was determined by ELISA. (**B**) Serum LPA level in normoglycemic and hyperglycemic HCC patients was determined by ELISA. (**C, F**) Confocal images of the uptake of the macropinocytosis marker and the LG-EV. The uptake of DiO-LG-EV (50 μg/mL, 1 h) and Rhodamine B (RhoB)-dextran (4 mg/mL, 1 h) in LG- and HG-cultured HepG2 cells (**C**), and in HepG2 cells pretreated with LPA (40 μM, 1 h. **F**), was checked. EIPA (50 μM, 1 h) and WTM (0.5 μM, 1 h) were used to inhibit the uptake. Scale bar: 20 μm. (**D, E, G, H**) The uptake of dextran and LG-EV is depicted in Box-and-scatter plots. Total particles per cell area were analyzed from at least five fields. The fluorescent signal intensity was quantified and normalized to LG-cultured HepG2 cells. (**I**) LG- or HG-cultured HepG2 cells, and (**L**) LPA-pretreated (40 μM, 36 h) HepG2 cells were incubated with/without LG-EV (2 μg/mL, 24 h) followed by sorafenib treatment (4 μg/mL, 36 h). Cellular viability was determined by the CCK8 assay. (**J, M**) Apoptosis level after LG-EV and sorafenib treatments was determined by Annexin V/7-AAD assay, and flow cytometry data are shown. (**K, N**) Apoptosis variation among treatments is depicted in Box-and-scatter plots. Hyperglycemic mouse models were induced, and HCC cell-derived xenograft (CDX) models were further developed and received sorafenib treatment. (**O**) Serum LPA levels in normoglycemic and hyperglycemic mice were determined by ELISA. (**P**) Growth curves of HCC among different groups were recorded and analyzed. Images (**Q**) and weights of excised HCC tumors (**R**) from different groups were analyzed. n=3. Mean±SD, * *P* < 0.05, ** *P* < 0.01. ns: no significant difference. Student's *t*-test.

To investigate whether hyperglycemia promotes LPA production, LPA levels were measured in different cell culture conditions. ELISA analysis showed that LPA levels were significantly elevated in HG-cultured media (Fig. 2A). Consistently, analysis of patient blood samples showed higher LPA levels in hyperglycemic HCC patients (Fig. 2B), indicating that HG enhances LPA production in both HCC cells and tissues. To determine whether HG-induced LPA elevation drives macropinocytosis and EV uptake, LG-EVs were isolated and characterized. Western blotting and flow cytometry confirmed the expression of established EV markers (HSP70, TSG101, and CD63) in LG-EVs (Supplementary Fig. S2A, S2B). Nanoparticle tracking analysis and TEM confirmed the size distribution and morphology of LG-EVs (Supplementary Fig. S2C, S2D). Confocal imaging showed increased LG-EVs and dextran (a macropinocytosis marker) uptake in HG-cultured HCC cells (Fig. 2C-E, Supplementary Fig. S4A-C). Pretreatment with macropinocytosis inhibitors EIPA or wortmannin (WTM) reversed such enhanced uptake (Fig. 2C-E, Supplementary Fig. S4A-C), while CCK8 and flow cytometry assays confirmed that these inhibitor treatments did not affect cell viability or induce apoptosis at the tested concentrations (Supplementary Fig. S3). Similarly, LPA treatment increased LG-EV and dextran uptake in HCC cells, which was reversed by EIPA or WTM pretreatment (Fig. 2F-H, Supplementary Fig. S4D-F). Further treating HCC cells with ATX inhibitor (GLPG1690) showed that restraining endogenous LPA production significantly reversed exogenous LPA-stimulated macropinocytosis of LG-EVs (Supplementary Fig. S4G-J). Collectively, these findings suggest that HG-induced LPA elevation promotes EV uptake via macropinocytosis in HCC cells.

Next, we investigated whether HG- or LPA-induced LG-EV uptake contributes to SFR. Compared to LG-cultured HCC cells, HG-cultured cells exhibited reduced survival and increased apoptosis following sorafenib treatment. However, supplementation with LG-EVs rescued survival and reduced apoptosis in HG-cultured cells (Fig. 2I-K, Supplementary Fig. S5A-C), suggesting that HG-induced EV uptake enhances SFR. Consistently, LPA pretreatment increased HCC cell survival and reduced apoptosis under sorafenib treatment, and this effect was further enhanced by LG-EV supplementation (Fig. 2L-N, Supplementary Fig. S5D-F). These results indicate that LPA-induced EV uptake promotes SFR of HCC cells. To confirm these findings *in vivo*, we established hyperglycemic HCC xenograft models in nude mice. As expected, body weights differed between hyperglycemic and normoglycemic groups (Supplementary Fig. S6). ELISA analysis confirmed that serum LPA levels were elevated in hyperglycemic mice regardless of sorafenib treatment (Fig. 2O).

Tumor growth analysis revealed that HCC in hyperglycemic mice grew faster than in normoglycemic controls, even under sorafenib treatment (Fig. 2P). Consistently, xenograft tumors from hyperglycemic mice were larger and heavier, independent of treatment (Fig. 2O-R). Together, these results suggest that HG-induced LPA elevation enhances EV uptake and promotes sorafenib resistance in HCC.

### Macropinocytosis-mediated LG-EV uptake promotes SFR of HCC cells

**Figure 3:**
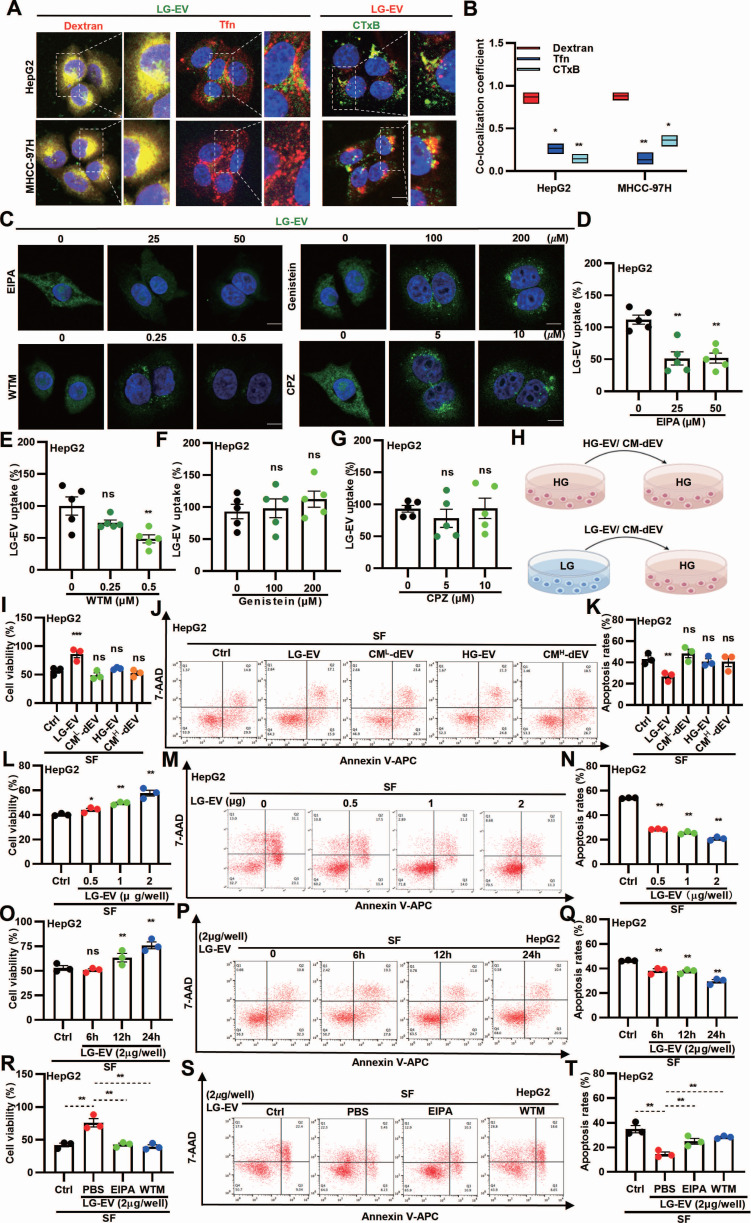
LG-EV uptake in HCC cells is mediated by macropinocytosis and promotes SFR. (**A**) Confocal images of the uptake of DiO-LG-EV (50 μg/mL, 1 h) colocalized with RhoB-dextran (4 mg/mL, 1 h) or AF647-Tfn (25 μg/mL, 1 h), and DiI-LG-EV (50 μg/mL, 1 h) colocalized with FITC-CTxB (10 μg/mL, 1 h) in HepG2 and MHCC-97H cells. Scale bar: 20 μm. (**B**) Co-localization coefficient between internalized LG-EV and endocytosis markers is determined. (**C**) Confocal images of DiO-LG-EV uptake (50 μg/mL, 1 h) in HepG2 cells pretreated with different dosages of endocytosis inhibitors for 1 h. Scale bar: 20 μm. (**D-G**) LG-EV uptake after different pretreatments is depicted in Box-and-scatter plots. Total particles per cell area were evaluated from at least five fields. (**H**) Diagram illustrating the transfer of HG- or LG-EV, or EV-depleted condition medium (CM-dEV) into HG-cultured HCC cells. HepG2 cells were pre-cultured with EV (2 μg/well, 24 h) or CM-dEV followed by sorafenib treatment (4 μg/mL, 36 h). (**I**) Cell viability was determined by the CCK8 assay. (**J**) Apoptosis level was determined by Annexin V/7-AAD assay, and flow cytometry data are shown. (**K**) Apoptosis variation among treatments is depicted by Box-and-scatter plots. HepG2 cells were pre-cultured with different concentrations of LG-EV for 24 h, followed by sorafenib treatment (4 μg/mL, 36 h). (**L, M, N**) Cellular viability and apoptosis variation were determined and depicted as mentioned above. HepG2 cells were pre-cultured with LG-EV (2 μg/well) for different time points, followed by sorafenib treatment (4 μg/mL, 36 h). (**O, P, Q**) Cellular viability and apoptosis variation were determined and depicted. HepG2 cells were pre-cultured with LG-EV (2 μg/well) and EIPA (50 μM) or WTM (0.5 μM) for 24 h and then received sorafenib treatment (4 μg/mL, 36 h). (**R, S, T**) Cellular viability and apoptosis variation were determined and depicted. All data are presented as means ± SEM. * *P* < 0.05, ** *P* < 0.01. ns: no significant difference. Student's *t*-test.

As CME, CDE, and macropinocytosis are the main pathways for EV uptake 16, we next investigated the endocytic pathway of LG-EVs in HCC cells. Confocal imaging revealed that internalized LG-EVs predominantly co-localized with dextran but minimally overlapped with the CME and CDE markers transferrin (Tfn) and cholera toxin B (CTxB), respectively (Fig. 3A-B). Consistently, pretreating HCC cells with endocytosis inhibitors showed that EIPA and WTM markedly inhibited LG-EV uptake in a dose-dependent manner, whereas chlorpromazine (CPZ; CME inhibitor) and genistein (CDE inhibitor) had no significant effect (Fig. 3C-G). These observations suggest that macropinocytosis mediates LG-EV uptake in HCC cells.

To further investigate the functional role of EV uptake in SFR, HCC cells were pre-incubated with EVs derived from HG or LG conditions (HG-EVs, LG-EVs) or with EV-depleted conditioned medium (CM-dEV) (Fig. 3H). CCK8 assays revealed that pre-incubation with LG-EVs, but not HG-EVs, enhanced cell viability under sorafenib treatment, whereas CM-dEVs failed to exert such an effect (Fig. 3I, Supplementary Fig. S7A). In parallel, flow cytometry showed that LG-EVs, but not HG-EVs, reduced sorafenib-induced apoptosis, while CM-dEVs had little impact (Fig. 3J-K, Supplementary Fig. S7B-C). Increasing the concentration of LG-EVs further enhanced cell viability and suppressed apoptosis (Fig. 3L-N, Supplementary Fig. S8A-C). Likewise, prolonged exposure to LG-EVs yielded similar, time-dependent effects (Fig. 3O-Q, Supplementary Fig. S8D-F). Together, these findings indicate that LG-EV uptake promotes SFR in a concentration- and time-dependent manner. Since macropinocytosis mediates LG-EV uptake, we next examined whether this pathway contributed to SFR. Both CCK8 and flow cytometry analyses confirmed that LG-EV pre-incubation increased viability and reduced apoptosis in sorafenib-treated HCC cells, whereas these effects were abolished by EIPA and WTM (Fig. 3R-T, Supplementary Fig. S9A-C). Collectively, these results demonstrate that macropinocytosis-mediated LG-EV uptake promotes SFR of HCC cells.

### LG-EV macropinocytosis is driven by LPA-induced Arf6 activation and confers SFR

**Figure 4:**
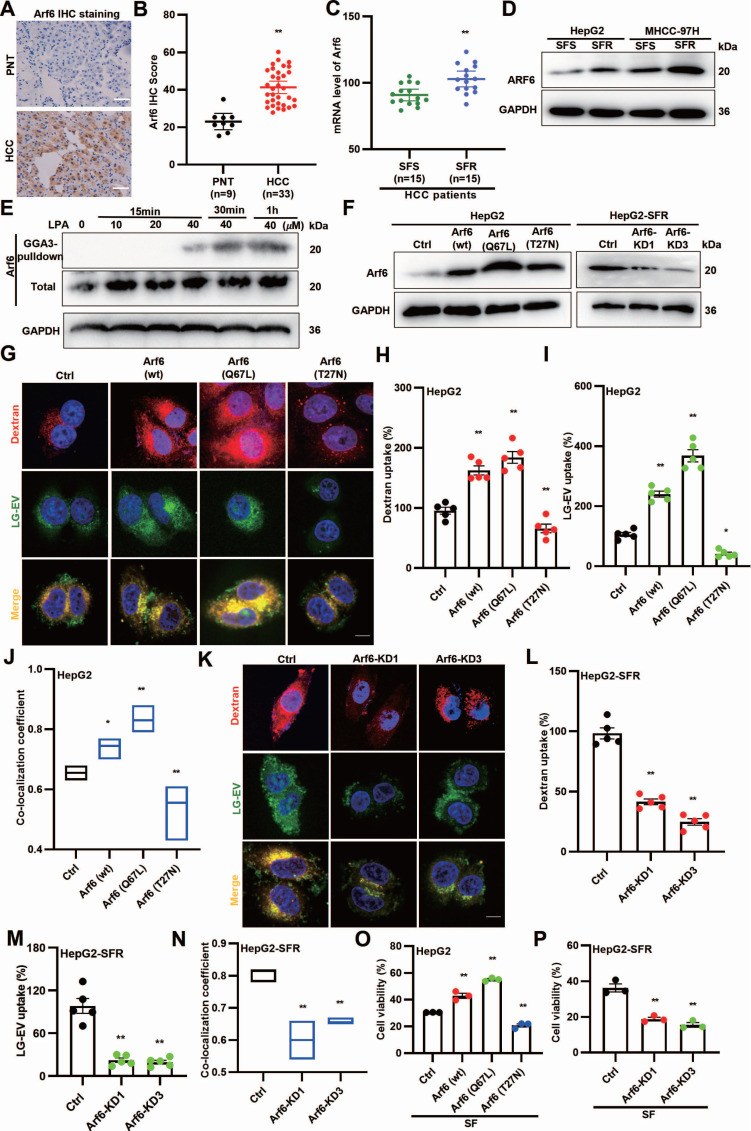
LPA-induced Arf6 activation drives LG-EV macropinocytosis and confers SFR of HCC cells. (**A**, **B**) IHC staining (**A**) and quantitative data (**B**) of PNT and HCC tissues. Scale bars: 100 µm. (**C**) mRNA level of Arf6 in SFR- and SFS-HCC tissues. Data are derived from the GEO database (GSE109211). (**D**) Arf6 expression level in SFR- and SFS-HCC cells was determined by Western blotting. (**E**) HepG2 cells were treated with different concentrations of LPA, and Arf6 activation level at various time points was determined by Western blotting. (**F**) Arf6-OE or -KD level in lentivirus-transduced cells was determined by Western blotting. (**G**) Confocal imaging of the DiO-LG-EV (50 μg/mL, 1 h) and RhoB-dextran (4 mg/mL, 1 h) uptake in Arf6-OE HepG2 cells. Scale bar: 20 μm. (**H-I**) LG-EV and dextran uptake in Arf6-OE HepG2 cells is depicted in Box-and-scatter plots. Total particles per cell area were evaluated from at least five fields. (**J**) The co-localization coefficient between internalized LG-EVs and dextran was analyzed in control, Arf6(wt), Arf6(Q67L), and Arf6(T27N) cells. (**K**) Confocal imaging of the DiO-LG-EV and RhoB-dextran uptake in Arf6-KD HepG2 cells. Scale bar: 20 μm. (**L-M**) LG-EV and dextran uptake and their co-localization coefficient in Arf6-KD HepG2 cells are depicted and analyzed. Cell viability of Arf6-OE (**O**) and Arf6-KD HepG2 cells (**P**) after sorafenib treatment (4 μg/mL, 36 h) was determined by the CCK8 assay. All data are presented as means ± SEM. * *P* < 0.05, ** *P* < 0.01. Student's *t*-test.

Since LPA promotes LG-EV macropinocytosis and SFR, and Arf6 activation drives macropinocytosis 18, we hypothesized that LPA-induced Arf6 activation facilitates LG-EV macropinocytosis and contributes to SFR. To determine the expression profile of Arf6, HCC surgical specimens were collected for analysis. IHC staining revealed higher Arf6 protein levels in HCC tissues (Fig. 4A-B). Consistently, GEO dataset analysis showed elevated Arf6 transcription in SFR-HCC samples (Fig. 4C). Western blotting further confirmed that Arf6 protein levels were increased in SFR-HCC cells (Fig. 4D). These results indicate that Arf6 is highly expressed in SFR-HCC tissues and cells. We next tested whether LPA activates Arf6 in HCC. GGA-based GST pull-down assays showed that LPA indeed induced Arf6 activation in a dose- and time-dependent manner (Fig. 4E). To determine whether Arf6-overexpression (OE) or constitutive activation could boost LG-EV macropinocytosis, HCC cells with stable Arf6-OE (wt, constitutively active Q67L, and dominant-negative T27N) and SFR-HCC cells with stable Arf6-knockdown (KD) were generated (Fig. 4F). Confocal imaging revealed that LG-EV and dextran uptake were markedly enhanced in Arf6-OE HCC cells, with the strongest effect observed in Arf6(Q67L)-OE HCC cells (Fig. 4G-I). Co-localization analysis further confirmed increased uptake and trafficking of LG-EVs with dextran in Arf6(wt)- and Arf6(Q67L)-OE HCC cells (Fig. 4J). In contrast, Arf6-KD or expression of dominant-negative Arf6(T27N) reduced LG-EV and dextran uptake as well as their co-localization (Fig. 4G-N). These findings suggest that Arf6-OE or constitutive activation enhances LG-EV macropinocytosis, while its knockdown or inactivation suppresses this process. Finally, functional assays demonstrated that sorafenib-suppressed viability was significantly alleviated in Arf6(wt)- and Arf6(Q67L)-OE HepG2 cells, but exacerbated in Arf6-KD and Arf6(T27N)-OE cells (Fig. 4O-P). Together, these results suggest that LPA-induced Arf6 activation drives LG-EV macropinocytosis and SFR of HCC cells.

### LG-TME increases FUT1-mediated CD147 fucosylation on EV surface

**Figure 5:**
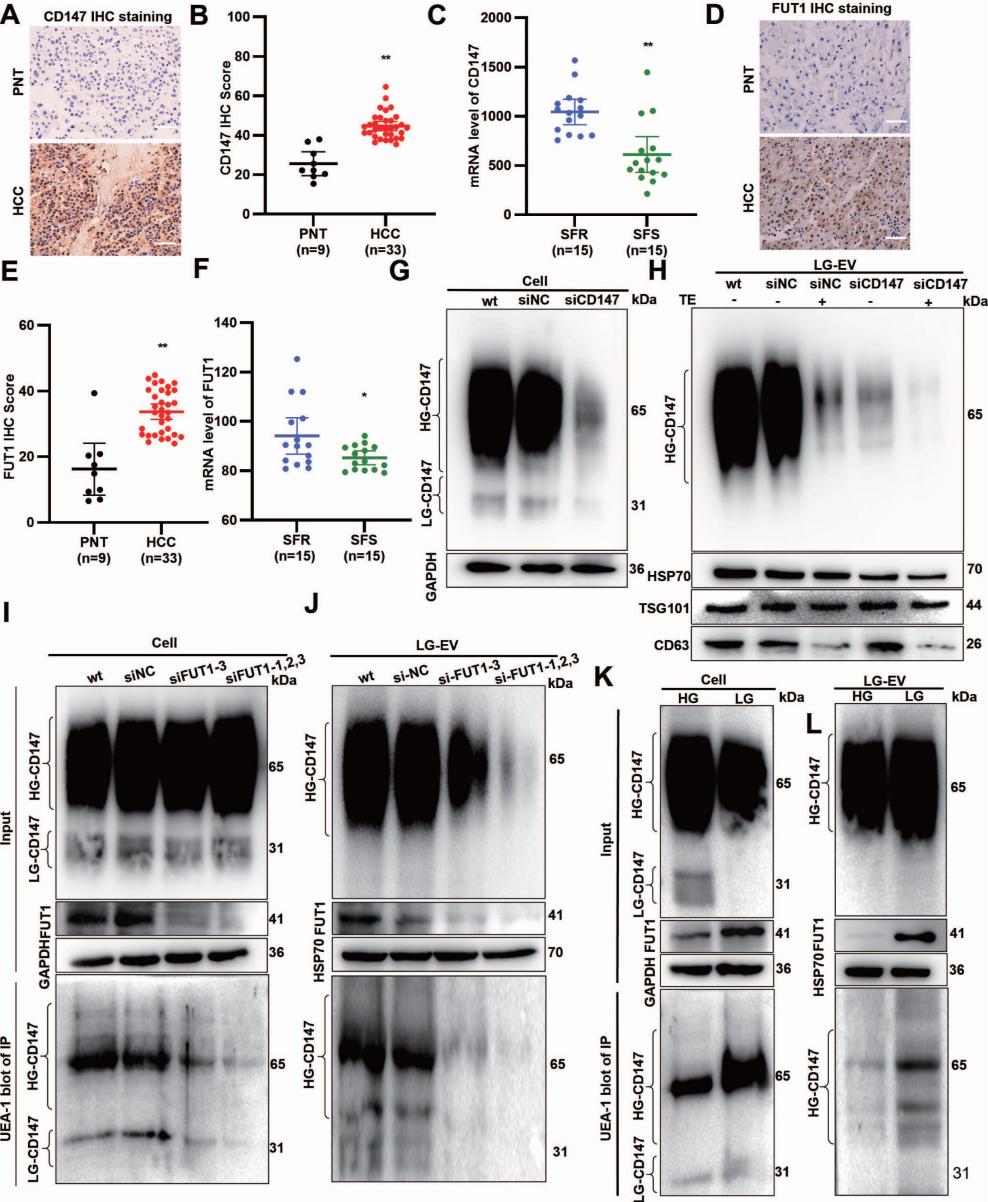
Glucose deprivation increases FUT1-mediated CD147 fucosylation on LG-EV surface. IHC staining (**A**) and quantitative data (**B**) of CD147 expression in PNT and HCC tissues. Scale bars, 100 µm. (**C**) mRNA level of CD147 in SFR- and SFS-HCC tissues. Data are derived from the GEO database (GSE109211). IHC staining (**D**) and quantitative data (**E**) of FUT1 expression in PNT and HCC tissues. Scale bars, 100 µm. (**F**) mRNA level of FUT1 in SFR- and SFS-HCC patients. Data are derived from the GEO database (GSE109211). Western blot analysis of the expression of CD147 and specific markers on LG-EV with or without trypsinization. CD147 expression in HepG2 cells (**G**) and LG-EV (**H**) was knocked down by siRNA transfection. The lysate of FUT1-KD HepG2 cells (**I**) and EV (**J**) was immunoprecipitated by anti-CD147 Ab, followed by UEA-1 blotting. UEA-1 blot determined the CD147-IP from HepG2 cells (**K**) and EV (**L**) under HG- and LG-conditions. * *P* < 0.05, ** *P* < 0.01. Student's *t*-test.

Multiple pieces of evidence indicate that CD147 overexpression (CD147-OE) promotes MDR in cancers, with FUT1 mediating CD147 fucosylation 6, 23. To determine the CD147 and FUT1 expression profile, HCC surgical specimens were collected. IHC staining revealed that both CD147 and FUT1 were significantly upregulated in HCC tissues (Fig. 5A, B, D, E). GEO database mining showed that these two proteins are highly expressed in SFR-HCC tissues (Fig. 5C, F).

To determine whether CD147 localizes on the LG-EV surface, we applied an RNAi-combined trypsin digestion approach. Western blotting showed that high-glycosylated CD147 (HG-CD147) and low-glycosylated CD147 (LG-CD147) were present in cell lysates, but only HG-CD147 was detected on LG-EVs (Fig. 5G, 5H, Supplementary Fig. S10A, S10B). CD147-KD reduced HG-CD147 levels in LG-EVs, while trypsin digestion completely removed it. Importantly, CD147-KD entirely abolished HG-CD147 on LG-EVs (Fig. 5H, Supplementary Fig. S10B), confirming that HG-CD147 is specifically displayed on the surface of HCC-derived LG-EVs.

Abnormal CD147 glycosylation has been reported to enhance HCC stemness, largely through FUT1-mediated fucosylation 6, 22, 27. To determine whether LG-EV-associated CD147 undergoes FUT1-dependent fucosylation, we performed immunoprecipitation (IP) followed by UEA-1 blotting. Fucosylated CD147 was detected in both cellular and LG-EV lysates. In FUT1-knockdown (FUT1-KD) HCC cells, CD147 fucosylation was markedly reduced in both compartments (Fig. 5I-J, Supplementary Fig. S10C-D). Moreover, HG-CD147 on LG-EVs was significantly diminished following FUT1 silencing, suggesting that FUT1 directly mediates CD147 fucosylation in HCC cells and on LG-EV surfaces (Fig. 5I-J, Supplementary Fig. S10C-D). Given that glucose deprivation induces aberrant CD147 fucosylation 6, we next examined whether LG culture conditions alter CD147 glycosylation on EVs. Western blotting showed that LG culture did not affect HG-CD147 levels in cell or LG-EV lysates but reduced LG-CD147 expression while markedly upregulating FUT1 (Fig. 5K-L, Supplementary Fig. S10E-F). Notably, IP-based UEA-1 blotting showed that LG culture strongly enhanced CD147 fucosylation in both HCC cells and LG-EVs (Fig. 5K-L, Supplementary Fig. S10E-F). These results suggest that LG culture elevates FUT1 expression, thereby promoting CD147 fucosylation on secreted LG-EVs.

### LG-EV macropinocytosis promotes SFR via CD147-fucosylation-mediated AKT/mTOR/4EBP1 signaling

**Figure 6:**
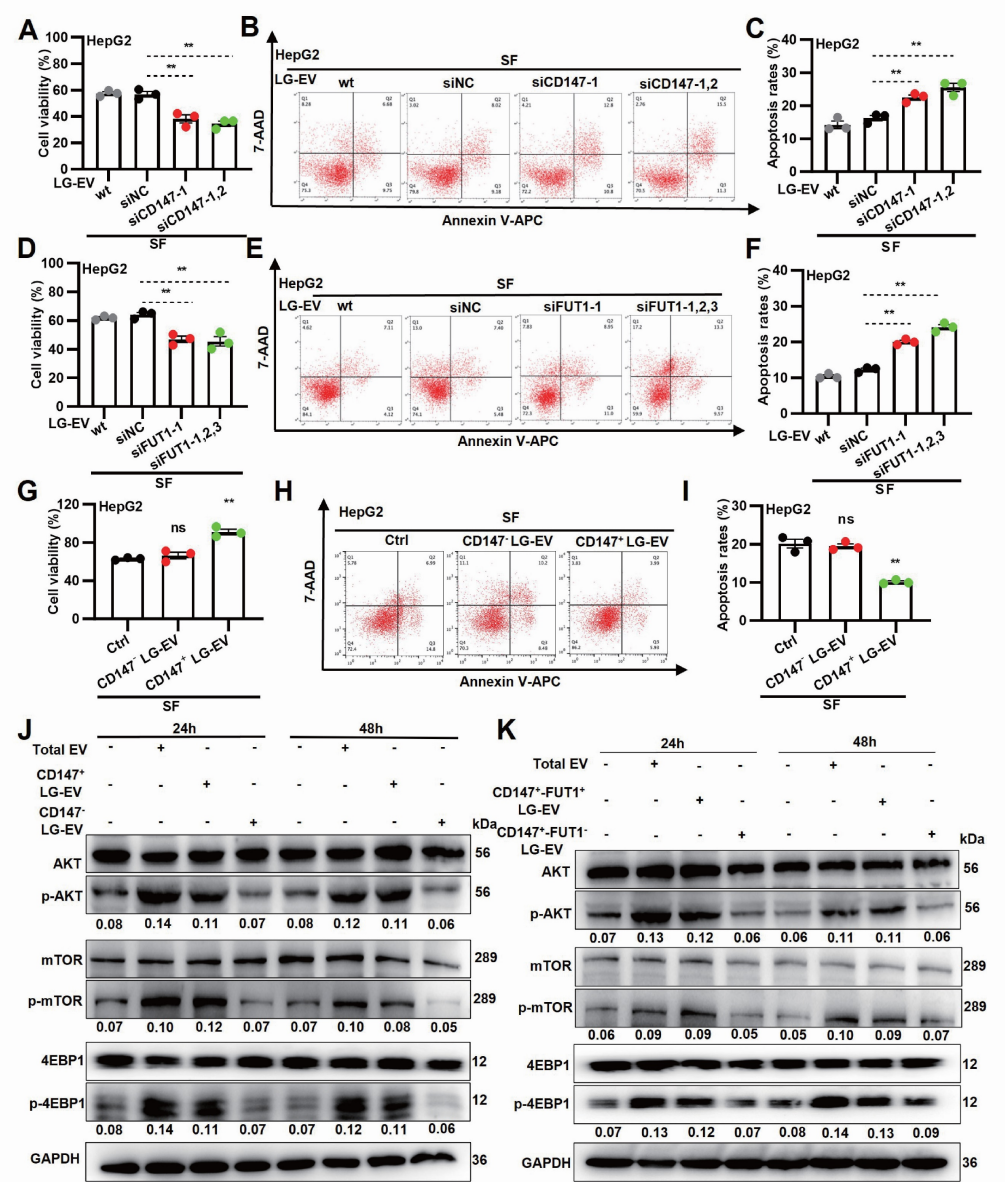
Macropinocytosis of CD147^+^ LG-EV promotes SFR via fucosylation-mediated AKT/mTOR/4EBP1 signaling axis. (**A, D**) Cellular viability was determined by the CCK8 assay. (**B, E**) Apoptosis level was determined by Annexin V/7-AAD-based flow cytometry assay, and the data are shown. (**C, F**) Apoptosis variation among treatments is depicted by Box-and-scatter plots. (**G, H, I**) Cellular viability and apoptosis variation were determined and depicted as mentioned above. Data are presented as means ± SEM. * *P* < 0.05, ** *P* < 0.01, ns: no significant difference. Student's *t*-test. (**J**) HG-cultured HepG2 cells were respectively treated with LG-EV, CD147^+^ LG-EV, and CD147^-^LG-EV (2 μg/well). The activated levels of *p*-AKT, *p*-mTOR, and *p*-4EBP1 in EV-treated HepG2 cells were determined by Western blotting. (**K**) HG-cultured HepG2 cells were treated with CD147^+^-FUT1^+^ LG-EV, CD147^+^-FUT1^-^ LG-EV (2 μg/well). Western blot determined the activation of *p*-AKT, *p*-mTOR, and *p*-4EBP1. Representative blotting and relative activation ratio are shown.

To determine whether EV macropinocytosis-promoted SFR is dependent on CD147 and FUT1 expression, LG-EVs were isolated from CD147- or FUT1-KD cells and co-cultured with HCC cells (Supplementary Fig. S11A). CCK-8 assays revealed that sorafenib-induced cell viability loss was further aggravated by incubation with CD147- or FUT1-KD LG-EVs (Fig. 6A, 6D, Supplementary Fig. S12A, S12D). In parallel, flow cytometry showed that these KD-EVs attenuated sorafenib-induced apoptosis in HCC cells (Fig. 6B, 6C, 6E, 6F, Supplementary Fig. S12B, 12C, S12E, 12F). These data suggest that SFR promoted by LG-EV macropinocytosis is dependent on FUT1-mediated CD147 fucosylation. To further examine the role of CD147 on LG-EV macropinocytosis-mediated SFR, CD147^+^ and CD147^-^ LG-EVs were isolated and co-cultured with HCC cells (Supplementary Fig. S11B). CCK-8 assays demonstrated that CD147⁺ LG-EVs, but not CD147⁻ LG-EVs, restored sorafenib-suppressed viability (Fig. 6G, Supplementary Fig. S13A). In parallel, flow cytometry confirmed that CD147⁺ EVs significantly suppressed sorafenib-induced apoptosis (Fig. 6H-I, Supplementary Fig. S13B-C). These results indicate that macropinocytosis of CD147^+^ LG-EVs promotes SFR of HCC cells. Given that FUT1 overexpression correlates with enhanced PI3K/AKT/mTOR signaling 6, we next examined whether fucosylated CD147⁺ EVs could activate this pathway. Western blotting revealed that HCC cells co-cultured with total EVs or CD147⁺ LG-EVs exhibited strong upregulation of *p*-AKT, *p*-mTOR, and *p*-4EBP1, whereas CD147⁻ EVs had little effect (Fig. 6J). To further confirm the requirement of FUT1-mediated fucosylation, FUT1⁺-CD147⁺ and FUT1⁻-CD147⁺ EVs were separately tested. Only FUT1⁺-CD147⁺ EVs robustly enhanced AKT/mTOR/4EBP1 activation (Fig. 6K). In addition, whether Arf6- and macropinocytosis-inhibition affected EV-production and stimulated signaling was also checked. Quantification, identification, and blotting results showed that blockage of Arf6-driven macropinocytosis did not affect the production of CD147⁺ LG-EVs, but significantly inhibited these EV-uptake-mediated activation of AKT/mTOR/4EBP1 signaling (Supplementary Fig. S14). These results suggest that LPA-activated Arf6-promoted CD147^+^ EV macropinocytosis promotes SFR via fucosylation-mediated AKT/mTOR/4EBP1 signaling.

### *In vivo* blocking Arf6-driven LG-EV macropinocytosis increases sorafenib therapy efficiency

**Figure 7:**
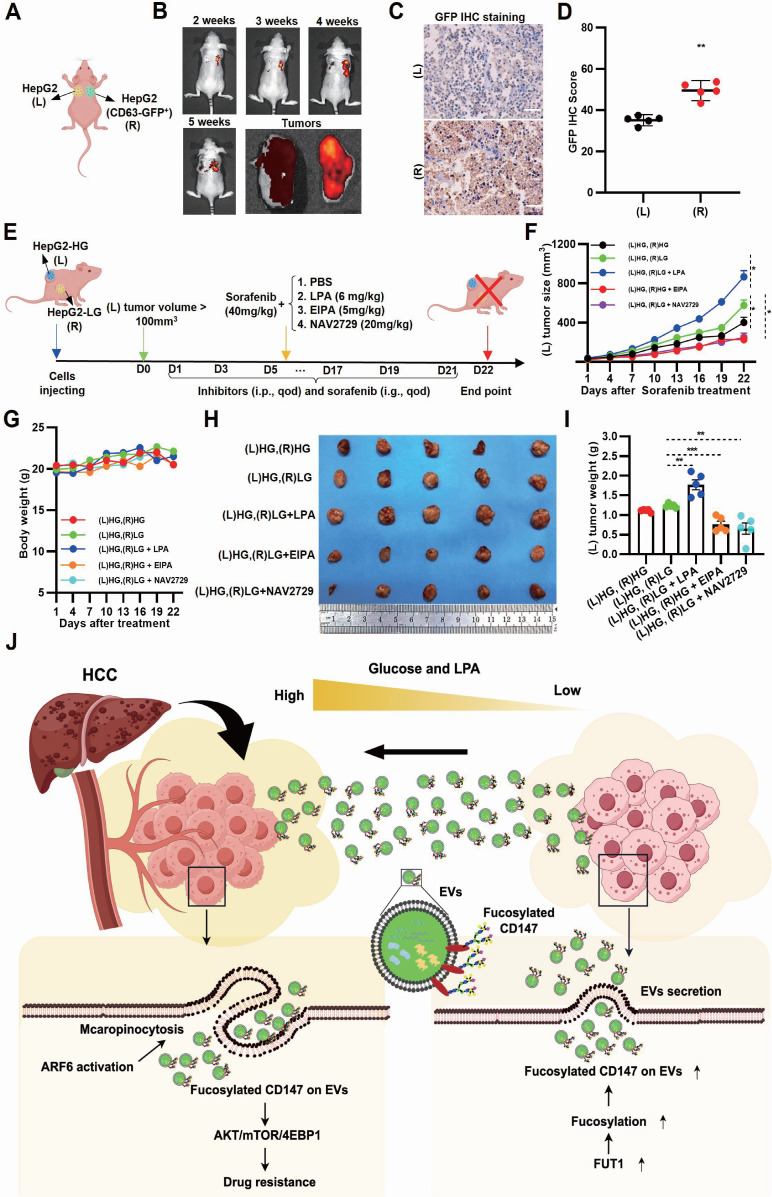
Inhibition of Arf6-driven LG-EV macropinocytosis increases sorafenib therapy efficiency in HCC-xenografted mouse models. (**A**) Diagram illustrating *in vivo* EV-transfer imaging. HepG2 cells with/without GFP-CD63 transfection were subcutaneously injected in nude mice at the right dorsal (R) and left dorsal (L) sides, respectively. The transfer of GFP^+^-EV was visualized by* in vivo* imaging. (**B**) Representative *in vivo* and *ex vivo* imaging of GFP signal in left and right HCC tumors from CDX models. IHC staining (**C**) and corresponding quantitative data (**D**) of bilateral HCC. Scale bars, 100 µm. (**E**) Diagram illustrating the therapeutic intervention of EV transfer in the Ng mouse model. Nude mice were subcutaneously injected with HG- and LG-cultured HepG2 cells at bilateral dorsal sides. HCC-xenografted mice were treated with sorafenib (40 mg/kg, i.g., qod, 21 days) together with macropinocytosis promoter LPA (6 mg/kg, i.p., qod, 21 days), or macropinocytosis inhibitor EIPA (5 mg/kg, i.p., qod, 21 days), or Arf6 inhibitor NAV2729 (20 mg/kg, i.p., qod, 21 days). (**F**) The volume of left-sided tumors (EV receiver) was monitored and analyzed. (**G**) The body weight of nude mice was monitored and analyzed. (**H**) Images of dissected HCC tissues from treated mice at day 22 post-administration. (**I**) Weight of excised HCC tissues from different groups. n=5. All data are presented as means ± SEM. ** *P* < 0.01. Student's *t*-test. (**J**) Working model: LPA-induced Arf6-driven macropinocytosis of CD147^+^ LG-EV promotes SFR of HCC via upregulating fucosylation-mediated AKT/mTOR/4EBP1 signaling.

To investigate whether EV transmission modulates sorafenib response *in vivo*, bilateral HCC xenografts were established in nude mice, with GFP-CD63 transfection in one lesion for EV tracing (Fig. 7A, Supplementary Fig. S15A). GFP imaging in excised HCC tissues revealed directional transfer of CD63-GFP⁺ EVs from the right to the left tumor (Fig. 7B, Supplementary Fig. S15B). IHC staining confirmed these results, indicating that GFP signal from transmitted EVs can be detected in tissue sections from the left HCC lesions (Fig. 7C-D). These results demonstrate that intertumoral EV transfer occurs *in vivo*. To further explore whether LPA-mediated, Arf6-driven EV macropinocytosis promotes SFR *in vivo*, LG-to-HG EV transmission models were developed. Bilateral xenograft mice were treated with the macropinocytosis inhibitor EIPA, Arf6 inhibitor NAV2729, or LPA as a macropinocytosis activator (Fig. 7E). Tumor volume monitoring showed that, under sorafenib treatment, left-side (EV-recipient) HCC grew faster in the (L)HG-(R)LG group compared with the (L)HG-(R)HG control group (Ctrl), indicating that EV transmission from LG- to HG-TME impairs sorafenib efficacy (Fig. 7F). Notably, LPA injection markedly accelerated the left-side (EV receiver) HCC growth (Fig. 7F), indicating LPA-mediated EV macropinocytosis promotes SFR. In contrast, EIPA or NAV2729 treatment slowed tumor growth on the left side (EV receiver) compared to Ctrl (Fig. 7F), confirming that Arf6-driven macropinocytosis promotes SFR. Endpoint measurements corroborated these findings: LPA markedly increased recipient tumor growth, whereas EIPA and NAV2729 significantly reduced it (Fig. 7H, I). Importantly, none of the treatments caused appreciable body weight loss (Fig. 7G). Together, these results suggest that LG-EV macropinocytosis promotes SFR *in vivo* through elevated LPA-Arf6 signaling, and that pharmacological inhibition of macropinocytosis or Arf6 can restore sorafenib sensitivity.

## Discussion

Glucose deficiency is a recognized TME feature of HCC. Indeed, rapid tumor growth, coupled with poor vascularization, results in reduced intratumoral glucose levels 5, 6. This condition is further aggravated by TACE, which induces ischemic starvation 42. In contrast, hyperglycemia is frequently observed in HCC patients with chronic viral infection (HBV, HCV) or metabolic disorders such as diabetes, MASLD, and MASH 43, 44. Periprocedural hyperglycemia is particularly concerning, as elevated blood glucose levels correlate with higher recurrence following ablation therapy 7, 45. Furthermore, TACE-induced hyperglycemia is closely associated with adverse clinical outcomes of HCC patients 7, 45, 46. These conditions establish a heterogeneous TME in HCC, with both glucose-deficient and hyperglycemic niches. One underappreciated but critical mechanism by which hyperglycemia promotes HCC progression is through LPA production. In this study, we found that hyperglycemia robustly enhances LPA levels across HCC cells, tumor tissues, and hyperglycemic mouse models (Fig. 2A-B; Fig. 2O). The precise mechanism remains unclear, but hyperglycemia-induced ATX upregulation provides a plausible explanation. ATX, mainly secreted by adipocytes, is elevated in diabetic patients 47, and we observed strong ATX expression in HCC tissues (Fig. 1D-H). Importantly, LPA levels correlated tightly with SFR in our study (Fig. 1I-M; Fig. 2P-R; Fig. 7H-I, Supplementary Table S2), consistent with previous evidence linking high LPA with enhanced metastatic potential 11, 12.

A key finding here is that hyperglycemia-driven LPA production enhances macropinocytosis of LG-EVs. While macropinocytosis is classically associated with nutrient-deprived TME 18, 48, we observed that HG conditions unexpectedly promoted macropinocytosis-mediated EV uptake in HCC cells (Fig. 2C-H). This aligns with prior reports showing: (1) hyperglycemia increases macropinocytosis in *Dictyostelium amoebae* 20, and (2) EVs are more efficiently internalized by immune cells from diabetic patients 49. Mechanistically, LPA activates small GTPases to drive malignant signaling 50. Our data indicate that LPA robustly activates Arf6, thereby stimulating macropinocytosis of LG-EVs in HCC cells (Fig. 2F-H; Fig. 4E-N). Three mechanisms may underlie this effect: (1) LPA binding to G-protein-coupled receptors (LPARs), which triggers EFA6 recruiting to GTP-Ga12, thus activating downstream Arf6 51; (2) Arf6 activation coordinates Rac1, Cdc42, and RhoA to initiate macropinosome formation 18, 52, and (3) LPAR1, 3, and 6 are highly expressed in HCC 53. Consistently, we observed elevated Arf6 expression in HCC, particularly in SFR-HCC (Fig. 4A-D). These results, in conjunction with the previously described mechanistic connections, provide additional evidence that LPA-induced and Arf6 activation-mediated LG-EV macropinocytosis contributes to the promotion of SFR in HCC.

Macropinocytosis is a well-established mechanism of MDR in cancers 16, 54, 55, yet few studies have linked it to EV uptake. Here, we demonstrate for the first time that macropinocytosis-mediated LG-EV uptake promotes SFR in HCC (Fig. 2I-N; Fig. 3I-T). Unlike prior studies implicating exosomes, microvesicles, or small EVs 56, 57, we identify a unique EV subtype, i.e., LG-EVs, whose macropinocytic uptake drives SFR of HCC (Fig. 1-7). Moreover, the decisive factor appears to be fucosylated CD147 on the EV surface (Fig. 5G, H; Fig. 6A-D; 6H-K), as both CD147 and EVs have recently been implicated in promoting HCC progression and drug resistance 16, 21, 22, 27, 58, 59. These observations differ from earlier findings where EVs conveyed miRNAs or essential metabolites to protect against sorafenib-induced apoptosis or ferroptosis 55, 56, 60. CD147 is recognized as a multifaceted driver of cancer progression, chemoresistance by affecting hyaluronan production, lactate efflux, and subcellular localization of ABCG2 23, and stemness via fucosylation 6. Our results strongly support that fucosylated CD147 on LG-EVs is a central mediator of SFR (Fig. 5I-L, Fig. 6E-G).

The mTOR signaling cascade plays a central role in tumor progression by regulating key processes such as cancer cell survival, apoptosis, autophagy, and angiogenesis. As an upstream regulator, AKT activates and modulates mTOR signaling. Once activated, the AKT-mTOR axis promotes protein synthesis by phosphorylating and inactivating translational repressors, including 4E-binding protein 1 (4EBP1) 61. Our data shows that macropinocytosis of CD147^+^ EVs activates AKT/mTOR/4EBP1 axis (Fig. 6J-K). This finding aligns with a previous report 6 but differs from EV uptake-induced signaling observed in other cancers, where pathways such as VEGF/AKT/eNOS/NO, AKT/mTOR/p70S6K, and NF-κB/β-catenin are engaged 58, 62. Recent studies have demonstrated that activated AKT/mTOR signaling is strongly linked to cancer stemness and drug resistance 63, 64. Moreover, HG-CD147 has been reported to regulate glucose metabolism and promote cell survival in cancers 6, 59. In line with these findings, it is reasonable that CD147⁺ EV-driven macropinocytosis activates AKT/mTOR/4EBP1 signaling to mediate SFR.

Since the discovery of EV uptake, only a limited number of strategies have been developed to visualize this process *in vivo* 16. Most approaches rely on labeling or tagging purified EVs *in vitro* and subsequently tracking their biodistribution after systemic injection. However, such administration of exogenous EVs does not faithfully recapitulate the endogenous EV uptake within cancer tissues. A few studies have managed to live-image tumor-derived EVs, capturing their behavior at metastatic sites or within lymph nodes 65, 66. To visualize these processes *in vivo*, we developed a bilateral xenograft model with GFP-CD63-labeled EVs, enabling real-time imaging EV trafficking between HG- and LG-TME (Fig. 7A-D, Supplementary Fig. S15). To our knowledge, this is the first *in vivo* imaging of EV uptake within TME. Using this system, we confirmed that LPA supplementation enhanced SFR, while inhibition of macropinocytosis or Arf6 suppressed it (Fig. 7F-I). Previous studies have shown that EIPA treatment suppresses EV macropinocytosis and consequently impairs tumor metastasis or chemosensitivity 54, 67. Together, these *in vivo* findings further support the idea that targeting Arf6-dependent EV macropinocytosis counteracts LPA-induced SFR.

Despite the promising findings of this study, several limitations remain. First, *in vivo* EV imaging requires further optimization. The current GFP⁺-EV imaging, based on a bilateral HCC cell-derived xenograft mouse model, only simulates intertumoral EV transfer (Fig. 7A-D, Fig. S15). Visualizing EV transmission within a single tumor focus remains a challenge. Second, although LPA supplementation validated its pro-SFR role (Fig. 1J, Fig. 2L-N, Fig. S5, Fig. 7E-I), definitive evidence requires ATX-knockout models. Third, clinical validation is currently limited by sample size (Supplementary Table S2); larger cohorts of HCC patients treated with sorafenib plus TACE are needed.

In summary, we reveal that TACE enhances the TME heterogeneity in HCC, establishing a hyperglycemic periphery and a glucose-deficient core, which facilitates EV trafficking. Peripheral hyperglycemia-induced LPA upregulates Arf6-driven macropinocytosis of fucosylated CD147^+^ LG-EVs, which activates AKT/mTOR/4EBP1 signaling to promote SFR (Fig. 7J). Our findings highlight the LPA-Arf6-EV macropinocytosis axis as a novel therapeutic target to overcome SFR of HCC.

## Supplementary Material

Supplementary figures and tables.
