# Supplementary material for: Lysophosphatidic acid-induced Arf6-driven macropinocytosis of CD147+ extracellular vesicles promotes sorafenib resistance of hepatocellular carcinoma
Source: Int J Biol Sci. 2026 Jan 1;22(1):220–38. doi: 10.7150/ijbs.125483 (PMC12681849; doi:10.7150/ijbs.125483)
Supplement: Supplementary file 1 — Supplementary figures and tables. [file ijbsv22p0220s1.pdf]

Supplementary Information

Table S1 Oligonucleotides used in this study.

Oligonucleotide name	Sequence
shRNA	
Arf6-KD-A1:	AAAAGGAAGGTGCTATCCAAAATTTGGATCCAAATTTTGGAT AGCACCTTCC
Arf6-KD-A3	AAAAGCTCACATGGTTAACCTCTAATTGGATCCAATTAGAGG TTAACCATGTGAGC
PCR primers	
Arf6-OE-forward	CATGGATCCGCCACCATGGGGAAGGTGCTATC
Arf6-OE-reverse	CGGCTAGCTTAAGATTTGTAGTTAGAGG
siRNA	
CD147-KD1	GUACAAGAUCACUGACUCUtt
CD147-KD2	GUUCUUCGUGAGUUCCUCtt
FUT1-KD1	AAAGGAUCUCUCAAGUCCGCGTT
FUT1-KD2	GCUACACCGUGGAAAGACUTT
FUT1-KD3	UCGAUGUUUUCUUUACACCAC

Table S2 Recurrent risk of clinical HCC patients with or without TACE.

	Non-TACE (n=13)		TACE (n=20)	
Characteristics	No, n (%)	Yes, n (%)	No, n (%)	Yes, n (%)
Male	11 (84.6)		16 (80)	
Female	2 (15.4)		4 (20)	
≤55	5 (38.5)		6 (30)	
>55	8 (61.5)		14 (70)	
HBV	8 (61.5)	5 (38.5)	5 (25)	15 (75)
Cirrhosis	6 (46.2)	7 (53.8)	10 (50)	10 (50)
Hyperglycemia	12 (92.3)	1 (7.7)	13 (65)	7 (35)
AFP > 400 (ng/mL)	11 (84.6)	2 (15.4)	18 (90)	2 (10)
ALB < 45 (g/L)	11 (84.6)	2 (15.4)	19 (95)	1 (5)
AST/ALT > 3	12 (92.3)	1 (7.7)	18 (90)	2 (10)
Ki-67 > 30%	10 (76.9)	3 (23.1)	15 (75)	5 (25)
CD34 (+)	10 (76.9)	3 (23.1)	10 (50)	10 (50)
Capsule invasion	10 (76.9)	3 (23.1)	15 (75)	5 (25)
Microvascular invasion	10 (76.9)	3 (23.1)	14 (70)	6 (30)
Satellitosis	11 (84.6)	2 (15.4)	18 (90)	2 (10)

HBV, hepatitis B virus; AFP, alpha-fetal protein; ALB, albumin; AST, aspartate aminotransferase; ALT, alanine aminotransferase.

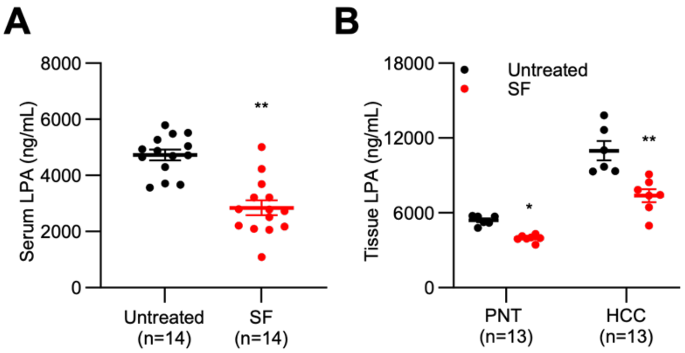

Fig. S1 Sorafenib (SRF) treatment decreases LPA levels in HCC patients. **(A)** Serum LPA levels of HCC patients with or without SRF treatment. **(B)** LPA levels of PNT and HCC tissues from patients with or without SRF treatment.

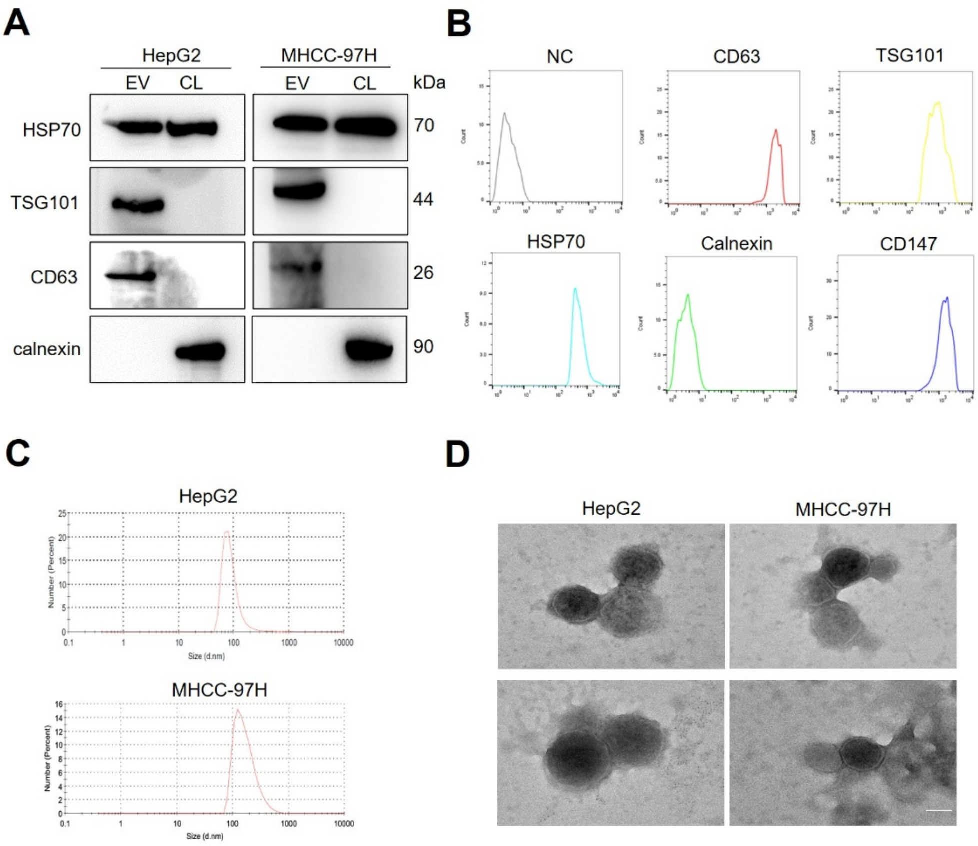

Fig. S2 Isolation and characterization of LG-EVs derived from cultured HCC cells. (**A**) Western blot analysis of the expression of EV markers in isolates from LG-cultured HepG2 and MHCC-97H cells. The EV-positive markers examined were HSP70, TSG101, and CD63. EV-negative marker: Calnexin. CL: cell lysate. (**B**) Flow cytometry analysis of the expression level of EV markers. (**C**) Size distribution analysis of isolated LG-EVs by Particle Metrix Zetaview nanoparticle tracking analyzer. (**D**) Morphology analysis of isolated LG-EVs by transmission electron microscopy (TEM). Scale bar: 50 nm.

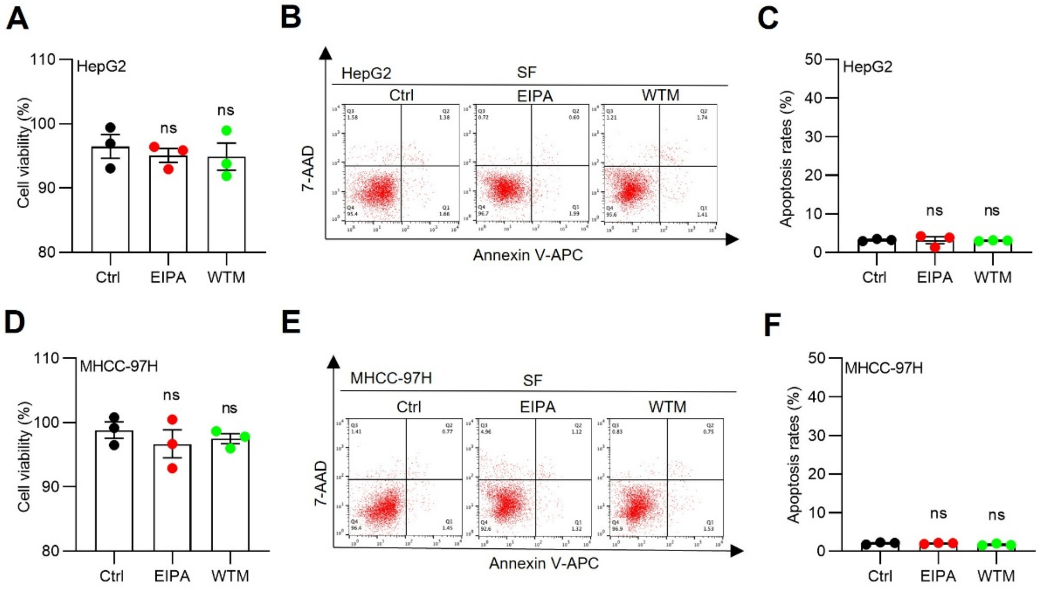

Fig. S3 Macropinocytosis inhibitors are not cytotoxic to HCC cells. (**A**, **D**) CCK8 assays were used to determine the viability of HepG2 (A) and MHCC-97H (D) cells pre-cultured with macropinocytosis inhibitors (EIPA: 50 μM. WTM: 0.5 μM). (**B**, **E**) Representative Annexin V/7-AAD-based flow cytometry data are shown. (**C**, **F**) Box-and-scatter plots depicted the apoptosis variation. All data are presented as means ± SEM. ns: no difference. Student's *t*-test.

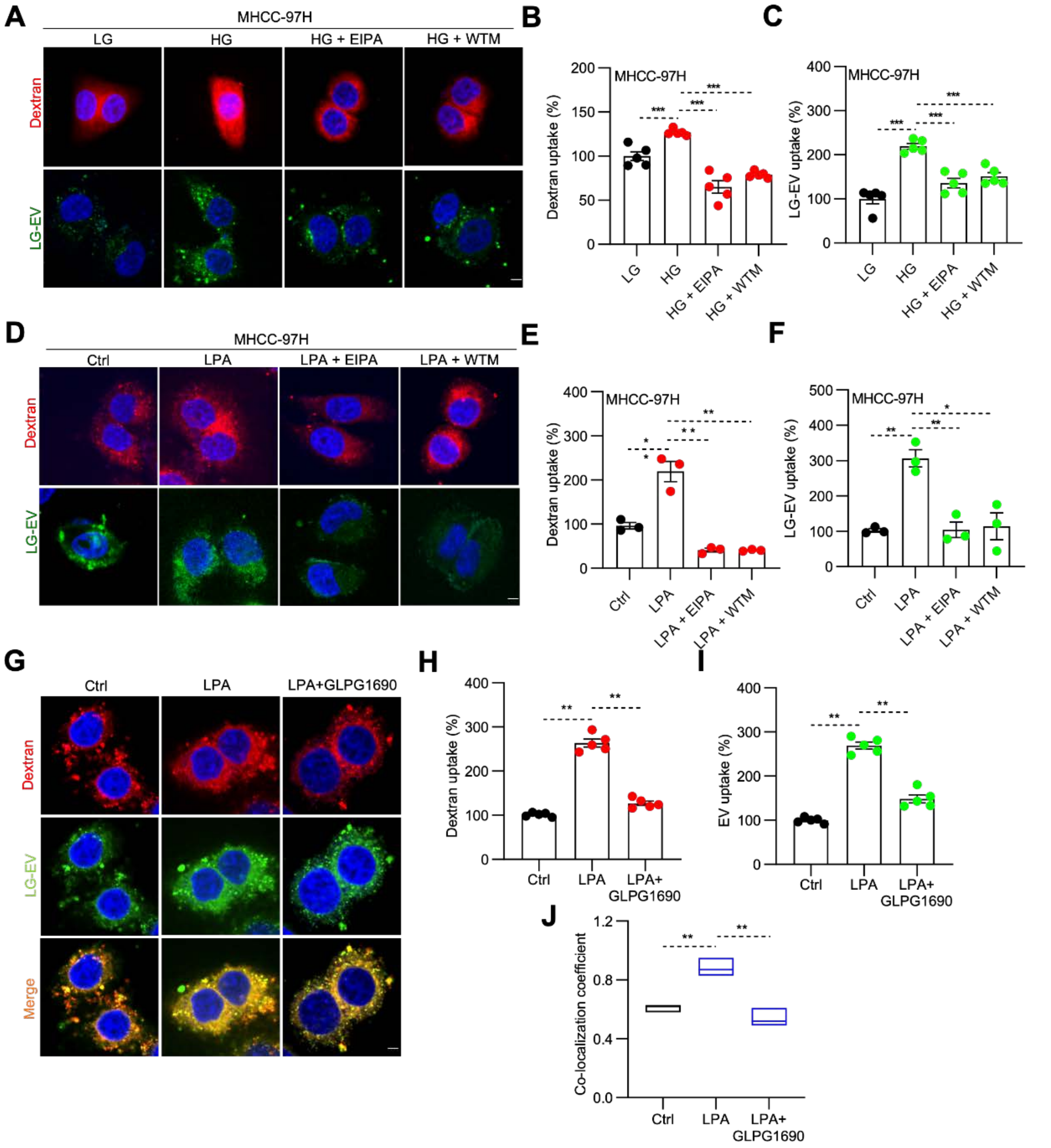

Fig. S4 High levels of glucose and LPA promote macropinocytosis and LG-EV uptake in MHCC-97H cells. Analysis of the uptake of DiO-LG-EV (50 μg/mL, 1 h) and RhoB-dextran (4 mg/mL, 1 h) in LG- or HG-cultured (**A**) or LPA-pretreated (**D**) MHCC-97H cells. EIPA (50 μM, 1 h) and WTM (0.5 μM, 1 h) were used to inhibit the uptake. (**A**, **D**) Representative confocal imaging of the uptake of dextran and LG-EVs. Scale bar: 20 μm. (**B**, **C**, **E**, **F**) The uptake of dextran and LG-EVs is depicted in Box-and-scatter plots. Total particles per cell area were analyzed from at least five fields. The fluorescent signal intensity was quantified and normalized to LG or Ctrl. LPA promotes the macropinocytosis of LG-EV, and GLPG1690 (ATX inhibitor) inhibits the macropinocytosis of LG-EV. **(G)** Confocal imaging of the DiO-LG-EV (50 μg/mL, 1h) and RhoB-dextran (4 mg/mL, 1h) uptake in HepG2 cells, respectively, treated with LPA (40 μM, 1h) and GLPG1690 (10 μM, 1h). Scale bar: 20 μm. **(H-I)** Dextran and LG-EV uptake in HepG2 cells is depicted in Box-and-scatter plots. **(J)** The co-localization coefficient between endocytic dextran and LG-EV was analyzed. All

data are presented as means ± SEM. * $P < 0.05$, ** $P < 0.01$, *** $P < 0.001$. Student's *t*-test.

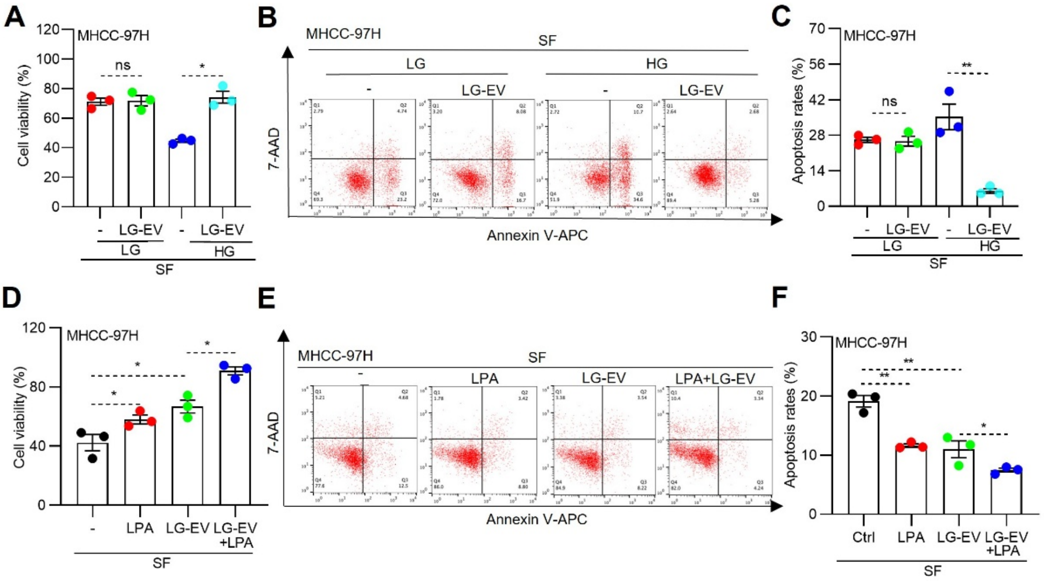

Fig. S5 HG- and LPA-induced LG-EV uptake promotes SFR of MHCC-97H cells. MHCC-97H cells were cultured in LG- or HG-condition (**A-C**), or pretreated with/without LPA (40 µM, 36 h. **D-F**). Next, cells were incubated with/without LG-EVs (50 µg/mL, 24 h), followed by sorafenib treatment (4 µg/mL, 36 h). (**A**, **D**) Cell viability was determined by the CCK8 assay (**B**, **E**). Apoptosis level was determined by Annexin V/7-AAD assay, and representative flow cytometry data are shown. (**C**, **F**) Apoptosis variation among treatments is depicted by Box-and-scatter plots. All data are presented as means ± SEM. * $P < 0.05$, ** $P < 0.01$. Student's *t*-test.

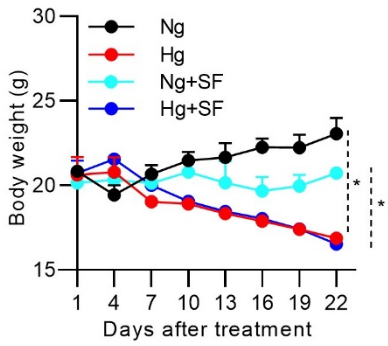

Fig. S6 Body weight curves of Ng- or Hg-mice treated with/without sorafenib. All data are presented as means ± SEM. * $P < 0.05$. Student's *t*-test.

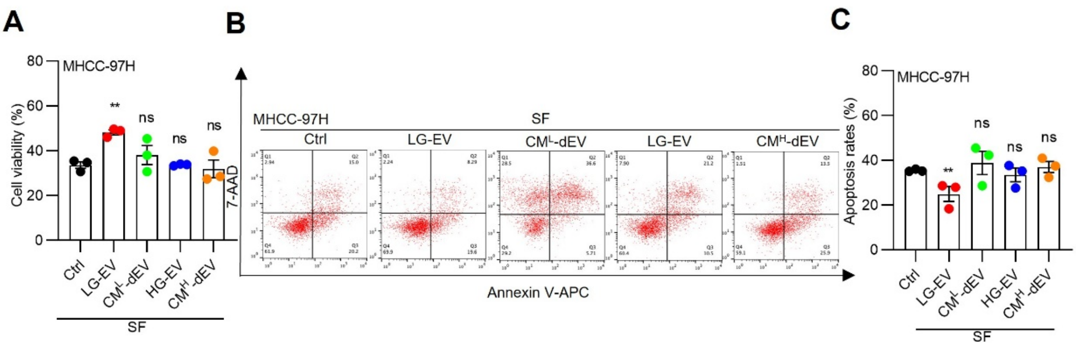

Fig. S7 The uptake of LG-EVs derived from MHCC-97H cells promotes SFR. MHCC-97H cells were pre-cultured with LG- or HG-EVs (2 μg/well, 24 h) or CM-dEV, followed by sorafenib treatment (6 μg/mL, 36 h). (**A**) Cell viability was determined by the CCK8 assay. (**B**) Apoptosis level was determined by Annexin V/7-AAD assay, and representative flow cytometry data are shown. (**C**) Apoptosis variation among treatments is depicted by Box-and-scatter plots. All data are presented as means ± SEM. ** $P < 0.01$, ns: no difference. Student's t-test.

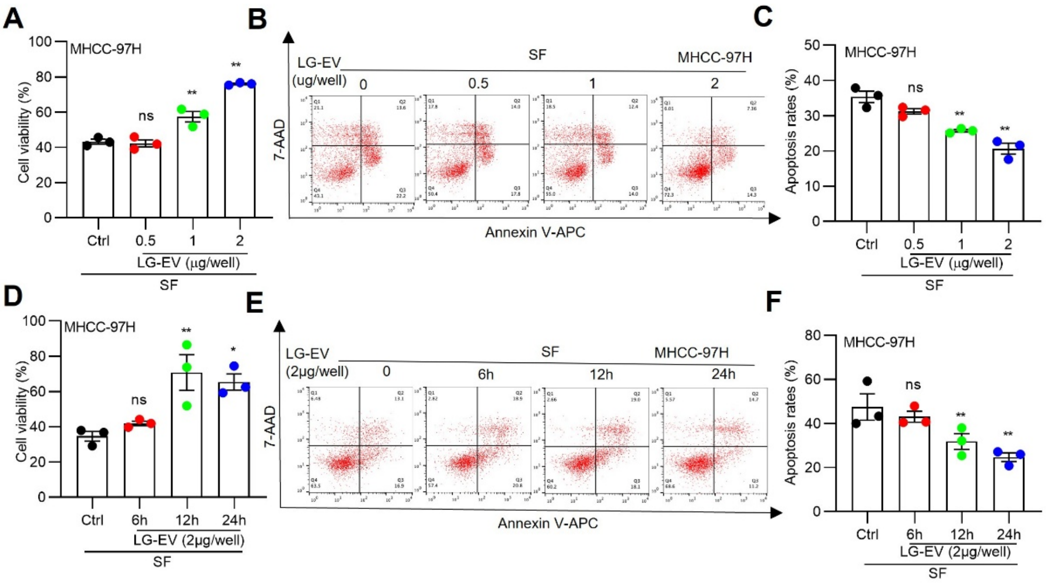

Fig. S8 The uptake of HCC-derived LG-EVs promotes SFR in a time- and concentration-dependent manner. MHCC-97H cells were pre-cultured with different concentrations (both for 24 h) or times (both at 2 μg/well) of LG-EVs, followed by sorafenib treatment (6 μg/mL, 36 h). (**A, D**) Cell viability was detected by the CCK8 assay. (**B, E**) Cell apoptosis level was determined by Annexin V/7-AAD-based flow cytometry assay, and representative data are shown. (**C, F**) Apoptosis variation among treatments is depicted by Box-and-scatter plots. All data are presented as means ± SEM. * $P < 0.05$, ** $P < 0.01$. ns: no difference. Student's t-test.

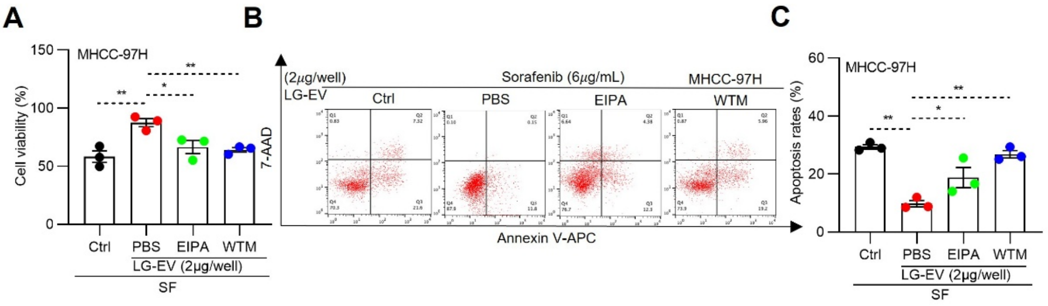

Fig. S9 Inhibition of LG-EVs' macropinocytosis decreases SFR of MHCC-97H cells. MHCC-97H cells were pre-cultured with LG-EV (2 μg/well) and EIPA (50 μM) or WTM (0.5 μM) for 24 h, and then received sorafenib treatment (6 μg/mL, 36 h). (**A**) Cell viability was determined by the CCK8 assay. (**B**) Cell apoptosis level was determined by Annexin V/7-AAD-based flow cytometry assay, and representative data are shown. (**C**) Apoptosis variation is depicted by Box-and-scatter plots. All data are presented as means ± SEM. * $P < 0.05$, ** $P < 0.01$. Student's *t*-test.

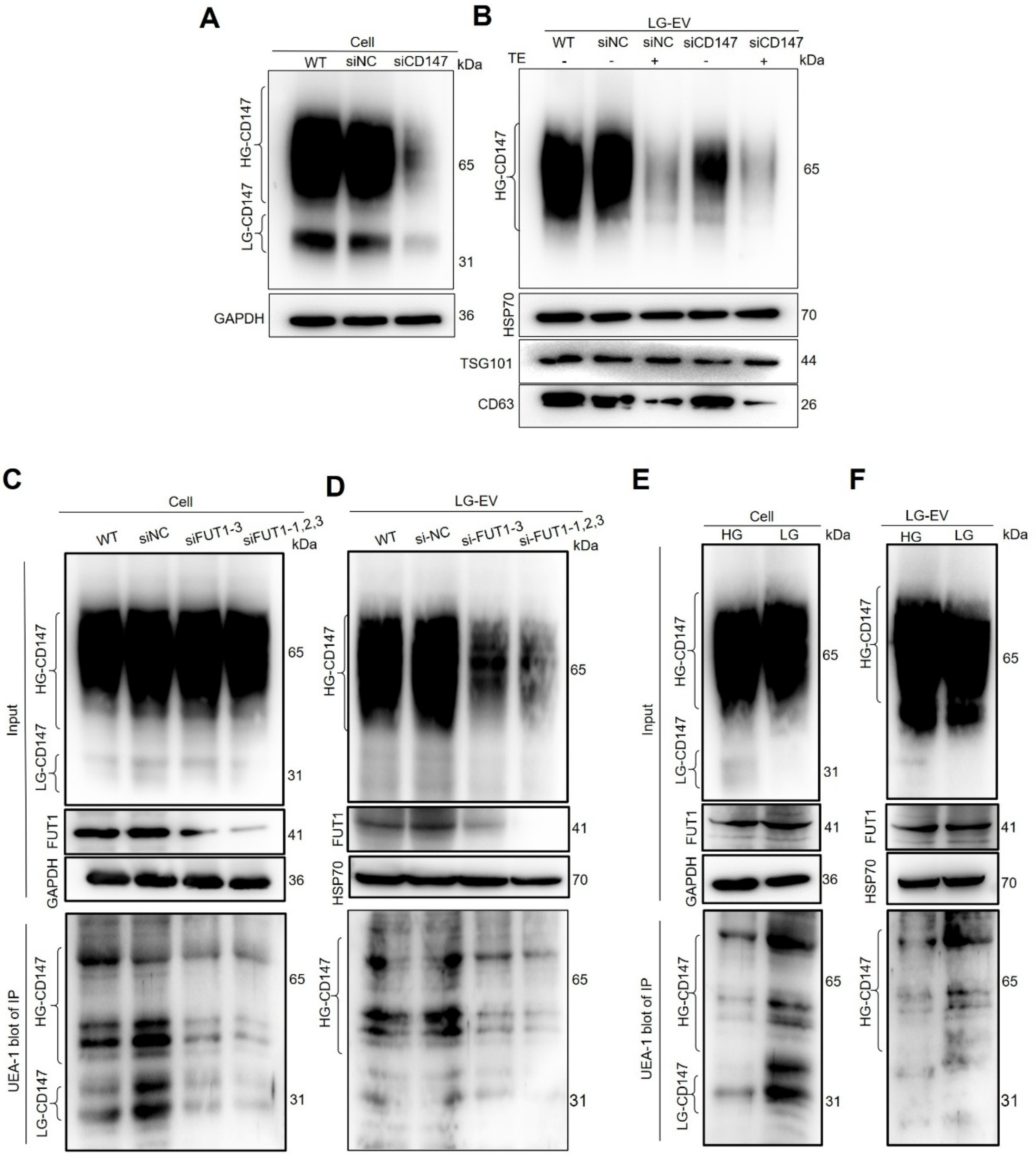

Fig. S10 Glucose deprivation increases FUT1-mediated CD147 fucosylation on MHCC-97H-derived EV surface. Western blot analysis of the expression of CD147 and specific markers on LG-EVs treated with or without trypsinization. CD147 expression in MHCC-97H cells (**A**) and LG-EVs (**B**) was knocked down by siRNA transfection. The lysate of FUT1-KD MHCC-97H cells (**C**) and LG-EVs (**D**) was immunoprecipitated by anti-CD147 Ab followed by UEA-1 blotting. Loading controls are GAPDH and HSP70, respectively. UEA-1 blot determined the CD147-immunoprecipitation from MHCC-97H cells (**E**) and LG-EVs (**F**) under HG- and LG-culture conditions.

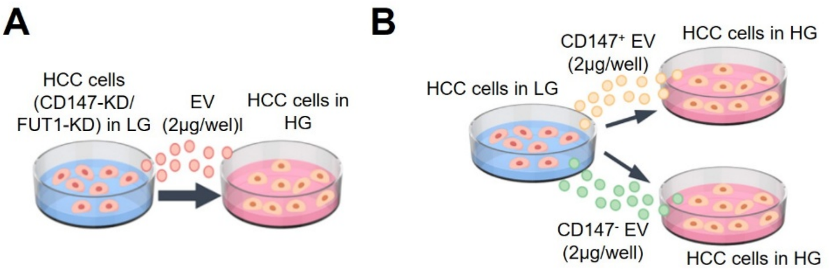

Fig. S11 (**A**) Diagram illustrating HG-cultured HCC cells incubated with LG-EV after CD147- or FUT1-KD. HepG2 cells were pretreated with CD147- or FUT1-KD LG-EV (2 ug/well) followed by sorafenib treatment (4 μg/mL, 36 h). (**B**) Diagram illustrating HG-cultured HCC cells incubated with CD147^{+} or CD147^{-} LG-EV. HepG2 cells were pretreated with CD147^{+} or CD147^{-} LG-EV (2 μg/well) followed by sorafenib treatment (4 μg/mL, 36 h).

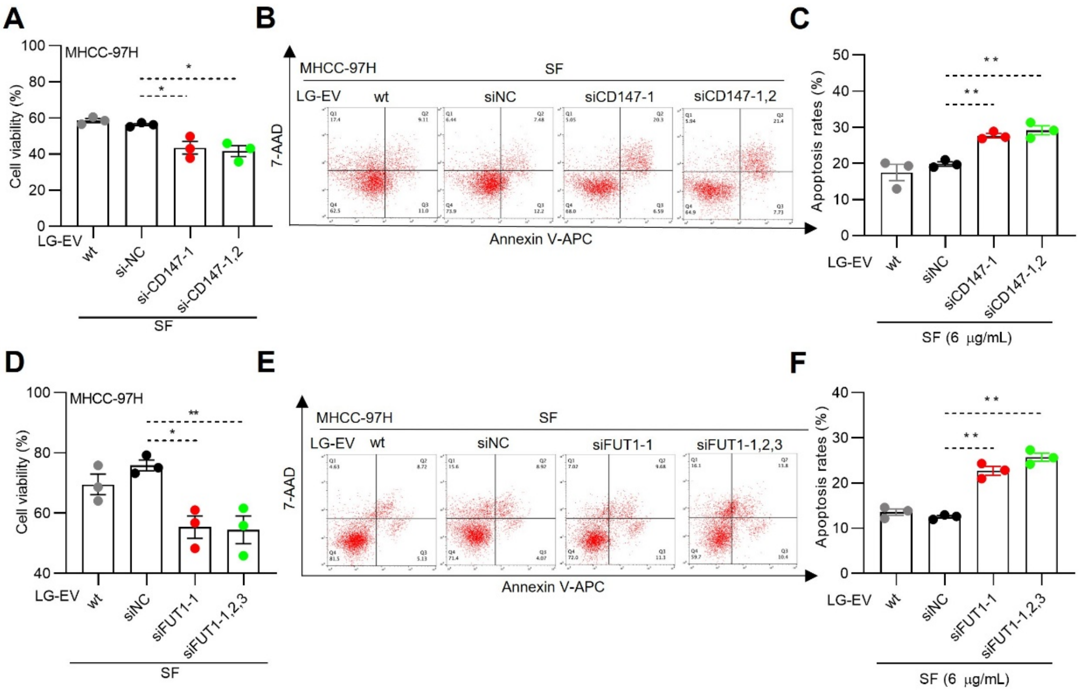

Fig. S12 Macropinocytosis of LG-EVs enhances SFR in MHCC-97H cells through CD147 and FUT1 expression. MHCC-97H cells were pretreated with CD147-KD or FUT1-KD LG-EVs (2 μg/well), followed by sorafenib treatment (6 μg/mL, 36 h). **(A, D**) Cellular viability was determined by the CCK8 assay. (**B, E**) Apoptosis level was determined by Annexin V/7-AAD-based flow cytometry assay, and representative data are shown. (**C, F**) Apoptosis variation among treatments is depicted by Box-and-scatter plots. All data are presented as means ± SEM. * $P < 0.05$, ** $P < 0.01$. ns: no difference. Student's *t*-test.

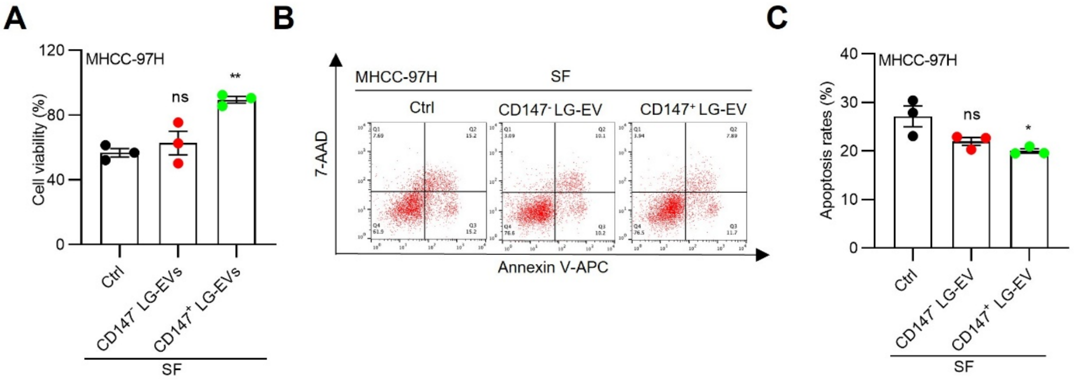

Fig. S13 Macropinocytosis of CD147+ LG-EV promotes SFR of MHCC-97H cells. MHCC-97H cells were pretreated with CD147+ or CD147- LG-EVs (2 μg/well), followed by sorafenib treatment (6 μg/mL, 36 h). (**A**) Cellular viability was determined by the CCK8 assay. (**B**) Apoptosis level was determined by Annexin V/7-AAD-based flow cytometry assay, and representative data are shown. (**C**) Apoptosis variation among treatments is depicted by Box-and-scatter plots. All data are presented as means ± SEM.* $P < 0.05$, ** $P < 0.01$. ns: no difference. Student's *t*-test.

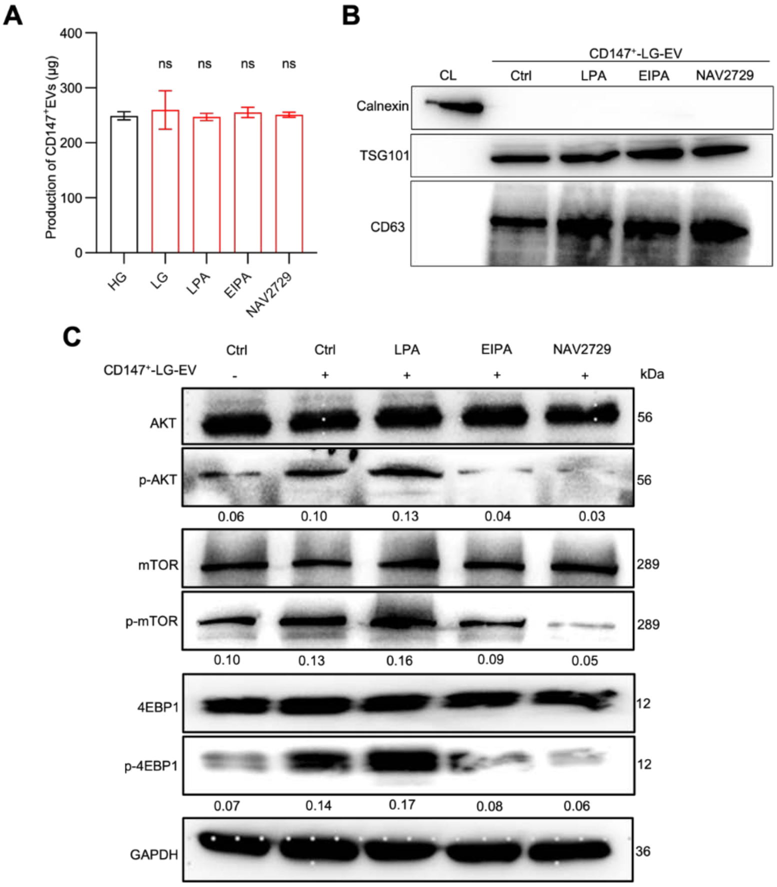

Fig. S14 LPA-induced Arf6-driven EV macropinocytosis does not affect the production of CD147+ LG-EVs but activates AKT/mTOR/4EBP1 signaling. **(A)** The quantification of CD147+ EVs using the BCA protein assay. **(B)** Western blot analysis of the expression of CD147+ EVs markers in

isolates from HepG2 cells treated with LPA (40uM, 1h), EIPA (50uM, 1h), and NAV2729 (5uM, 1h). **(C)** Western blot analysis of HepG2 cells cultured with CD147$^+$ LG-EV, which were pretreated with LPA (40uM, 1h), EIPA (50uM, 1h), and NAV2729 (ARF6 inhibitor, 5uM, 1h). The activated levels of p-AKT, p-mTOR and p-4EBP1 in HepG2 cells were determined by Western blotting.

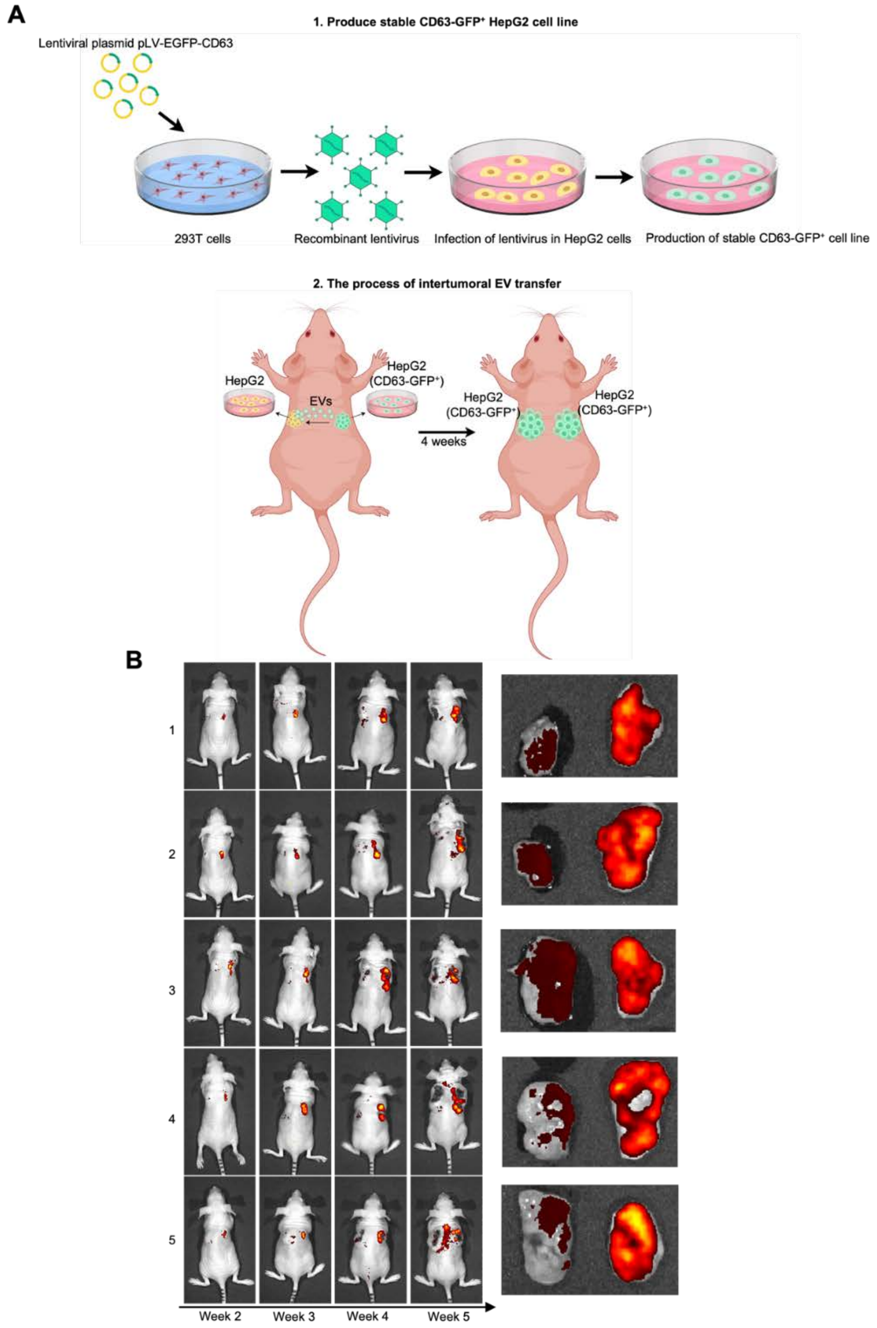

Fig. S15 ***In vivo* imaging of EV transfer. (A)** Schematic illustration of the *in vivo* EV-transfer imaging workflow. 1) Generation of stable CD63-GFP$^+$ HepG2 cells: the lentiviral plasmid pLV-EF1α-MCS-EGFP-CD63-IRES-puro was transfected into 293T cells to produce recombinant lentivirus; the collected viral supernatant was then used to infect HepG2 cells, followed by puromycin selection to establish a stable CD63-GFP$^+$ HepG2 cell line. 2) *In vivo* intertumoral EV

transfer: CD63-GFP^{+} HepG2 cells and untransfected HepG2 cells were subcutaneously injected into the right (R) and left (L) dorsal flanks of nude mice, respectively; EVs derived from CD63-GFP^{+} HepG2 cells were taken up by the contralateral (L) tumors, leading to detectable GFP fluorescence after 4 weeks. Tumor fluorescence was visualized by *in vivo* imaging. **(B)** *In vivo* and *ex vivo* images showing GFP fluorescence in right (R) and left (L) HCC xenograft tumors.
